# Enhanced Binding of Zn^2+^ Using a Sulfur Version of *o*-Aminophenol-Triacetate (APTRA): Introducing S-APTRA and Derivatives

**DOI:** 10.1021/acs.inorgchem.5c00275

**Published:** 2025-05-07

**Authors:** Christopher Hogg, Laura L. Duncan, David Parker, J. A. Gareth Williams

**Affiliations:** Department of Chemistry, Durham University, South Road, Durham DH1 3LE, U.K.

## Abstract

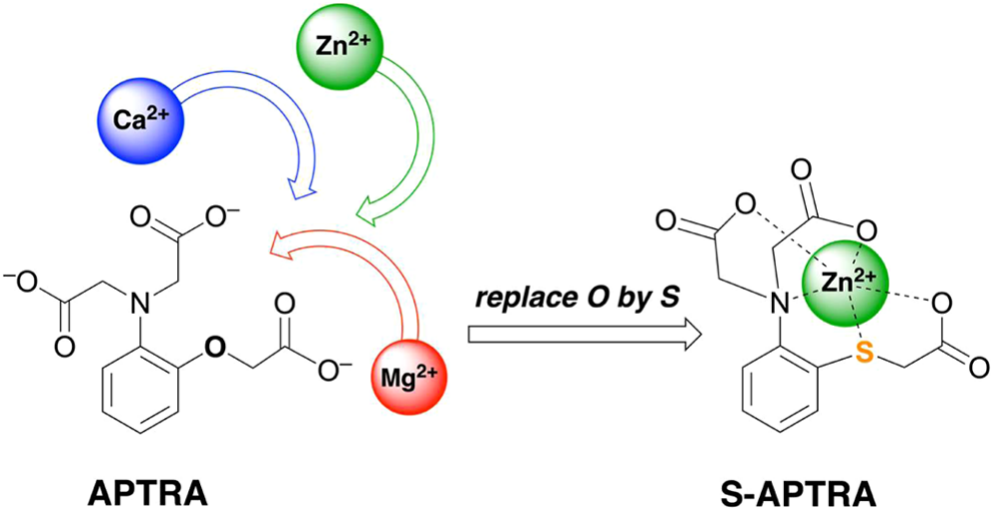

*Ortho*-aminophenol-*N,N,O*-triacetate (APTRA) is a pentadentate ligand adopted for the selective binding of Mg^2+^. It has been incorporated into fluorescent sensors for Mg^2+^, though it binds Ca^2+^ and Zn^2+^ more avidly. Here, the synthesis of a sulfur analogue of APTRA is reported, namely *ortho*-aminothiophenol-*N,N,S*-triacetate, referred to as S-APTRA. The binding of this new pentadentate ligand to Zn^2+^, Ca^2+^, and Mg^2+^ has been monitored in buffered aqueous solution by UV absorption spectroscopy. The replacement of the phenolic oxygen of APTRA by a sulfur atom renders S-APTRA capable of binding Zn^2+^ in a biologically relevant range (*K*_d_ = 6.6 ± 0.3 nM) with high selectivity over Mg^2+^ and Ca^2+^. The enhanced selectivity for Zn^2+^ is in line with the principles of “hard and soft acids and bases.” A tetradentate analogue omitting the S-appended carboxylate group, S-APDIA, is also reported. Its lower denticity leads to decreased affinity for Zn^2+^ (*K*_d_ = 8 ± 1 μM). The oxidation of S-APTRA and S-APDIA by *m*-CPBA leads to the sulfoxides SO-APTRA and SO-APDIA, which bind Zn^2+^ yet more weakly (*K*_d_ = 260 ± 20 mM and 3.6 ± 0.3 mM, respectively). This new family of ligands may prove appealing in the development of new carboxylate-based zinc sensors.

## Abstract 2

A sulfonated analogue of APTRA strongly binds Zn^2+^ in aqueous solution with high affinity over Ca^2+^ and Mg^2+^, offering a route to new carboxylate-based zinc sensors. The affinity for Zn^2+^ is attenuated for the corresponding sulfoxide ligand and for tetradentate analogues that omit the S-linked acetate group.

## Introduction

Zinc plays a crucial role in bioinorganic chemistry.^1^ It has a diverse range of structural roles in innumerable proteins, maintaining the conformations required for activity.^2^ It plays a key catalytic role in many enzymes, particularly hydrolytic ones, including critical systems like carbonic anhydrase that are ubiquitous across life forms.^3^ Detailed knowledge of the structure and function of zinc in such systems has been built up over the past 50 years or so, aided by protein crystallography and electron microscopy. On the other hand, the regulatory role of changes in the concentration of “free” Zn^2+^ is much less well understood.‡ Although values of [Zn^2+^]_free_ may be as low as the picomolar range in some biological systems,^4^ it is understood that the release of chelated Zn^2+^ can result in transient fluxes of the concentration of this ion.^5,6^ The view that changes in [Zn^2+^]_free_ have a role in cell signaling and regulation is increasingly accepted.^7,8^

**Figure 1:**
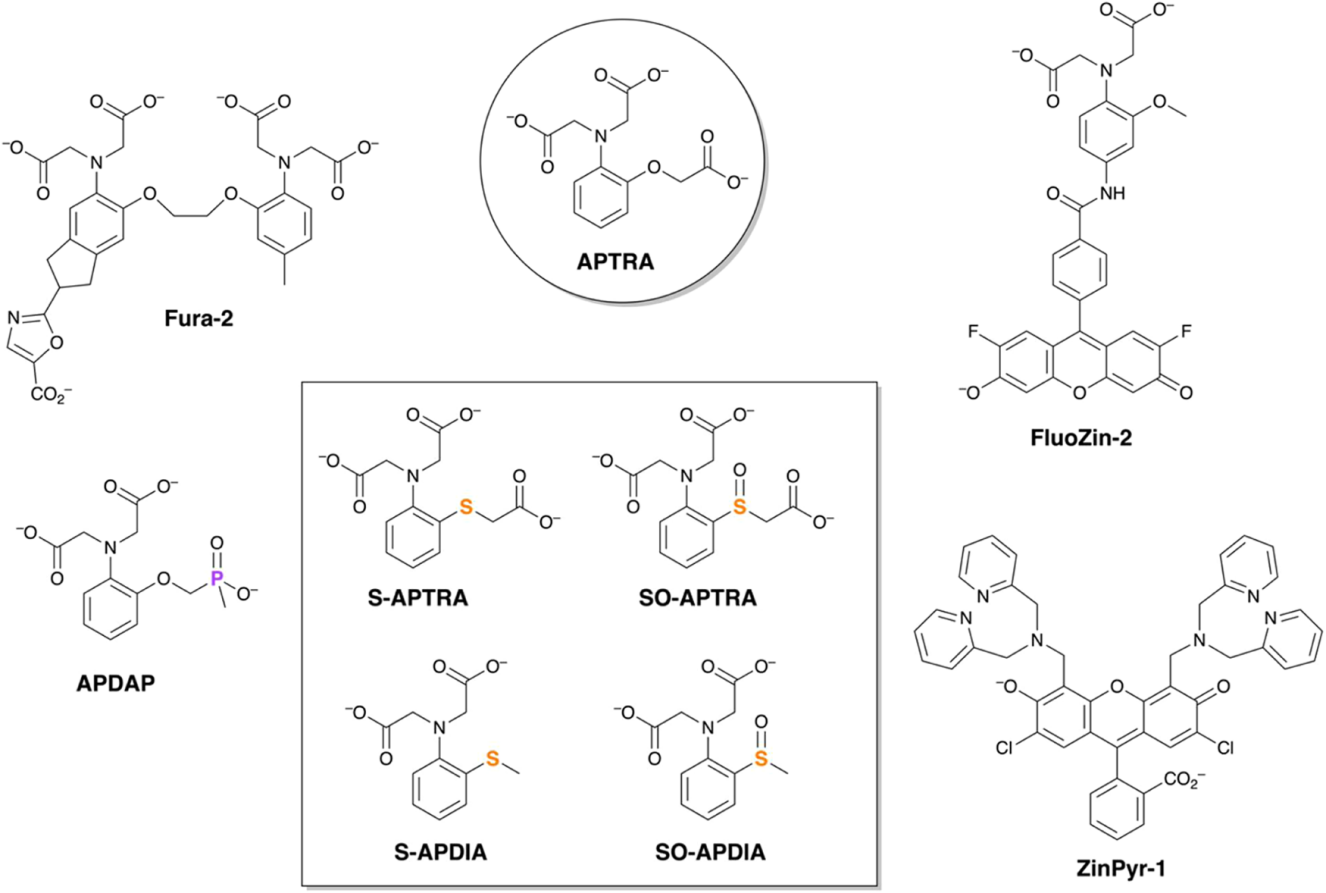
Structures of the four new sulfur-containing relatives of APTRA described in this work are shown in the square box. APTRA itself is circled. Fura-2 and FluoZin-2 incorporate the *ortho*-aminophenoldiacetate unit found in APTRA but omit the phenol-linked acetate; they have been deployed as fluorescent sensors for Ca^2+^ and Zn^2+^, respectively. APDAP is a phosphinate analogue of APTRA with enhanced selectivity for Mg^2+^. ZinPyr-1 is an example of a dipicolylamine-based sensor for Zn^2+^.

Key to furthering the understanding of the regulatory function of Zn^2+^ in biological systems is the ability to undertake real-time detection of changes in [Zn^2+^]_free_.^9^ The use of molecular fluorescent sensors in conjunction with fluorescence microscopy is attractive for this purpose, owing to the high sensitivity with which light can be detected, coupled with high spatial and temporal resolution.^10,11^ For Zn^2+^, early examples of such probes were based on 8-aminoquinoline.^12^ The development of fluorophores appended with the zinc-selective dipicolylamino (DPA) group, −N(CH_2_py)_2_ (Figure 1), by Lippard and Tsien subsequently led to further advances in monitoring [Zn^2+^]_free_.^13^ Others have successfully used such probes in more recent studies.^14^

Central to the function of such fluorescent sensors is the ligating unit, which should ideally bind the target cation with high selectivity over other potentially competing cations, and with a dissociation constant *K*_d_ under aqueous conditions that is comparable to the prevailing concentration of the target ion.^15^ Many DPA-based systems offer suitable affinities with *K*_d_ in the 1–10 nM range, but azacarboxylate-based ligands, such as those present in the FuraZin and FluoZin series of probes, often do not bind Zn^2+^ so strongly while suffering from competitive binding of Ca^2+^ and Mg^2+^.^16^ The development of carboxylate-containing ligands with high binding strengths and selectivity for Zn^2+^ would nevertheless be attractive, as they could benefit from facile cellular loading as their acetoxymethyl (AM) esters and from the greatly improved cellular retention following ester hydrolysis within the cell—strategies that are well-established from studies on Ca^2+^ sensors (e.g., Fura-2, Figure 1).^17^ Furthermore, problems with ternary complex formation—that is, where the Zn^2+^ ion is bound not only by the sensor but also to endogenous chelating moieties—have recently been highlighted for most of the currently available ligands,^18^ emphasizing the need to develop new chelates for Zn^2+^ sensors, including ones of higher denticity.

In this work, we report on a new family of ligands prepared through the modification of a well-known azacarboxylate ligand, *ortho*-aminophenoltriacetate, APTRA (Figure 1), that has been widely used for the detection of Mg^2+^, Ca^2+^, and indeed Zn^2+^.^19,20^ The key modification explored is the replacement of the phenol oxygen atom by a thiophenol sulfur atom, a change that is found to greatly enhance the selectivity of the resulting ligand (S-APTRA, Figure 1) for Zn^2+^ over the harder metal ions Ca^2+^ and Mg^2+^. We also discuss the properties of three closely related compounds, namely the sulfoxide analogue (SO-APTRA), and the corresponding compounds omitting the S-linked carboxylate unit (S-APDIA and SO-APDIA).

## Results and Discussion

### Molecular Design Strategy

Previously, we applied established principles of coordination chemistry to convert APTRA into a ligand that is more selective for Mg^2+^ over Ca^2+^ and Zn^2+^.^21^ In that case, our strategy was the replacement of the phenol-appended carboxylate by a methylphosphinate group, giving APDAP, which favors the binding of smaller, hard metal ions like Mg^2+^ (Figure 1).^22^ The present work was predicated on the notion that the substitution of the phenolic oxygen atom by sulfur would favor the binding of the softer Zn^2+^ ion over the harder Mg^2+^ and Ca^2+^, potentially leading to a high-denticity selective ligand, S-APTRA, for Zn^2+^ capable of operating under biologically relevant conditions. Meanwhile, the presence of the carboxylates could then offer potential advantages over DPA-based systems, such as enhanced water solubility and scope for cellular loading and accumulation using labile acetoxymethyl esters. Given the wide variation in [Zn^2+^]_free_ found across biological systems (pM to μM), we reasoned that simple changes to S-APTRA—such as oxidation to the sulfoxide or the omission of the thiol-linked acetate (*cf*. FuraZin-1 versus Mag-fura-2)—could plausibly offer access to lower-affinity Zn^2+^ probes suitable for use in more Zn^2+^-rich environments.

### Synthesis

**Scheme 1:**
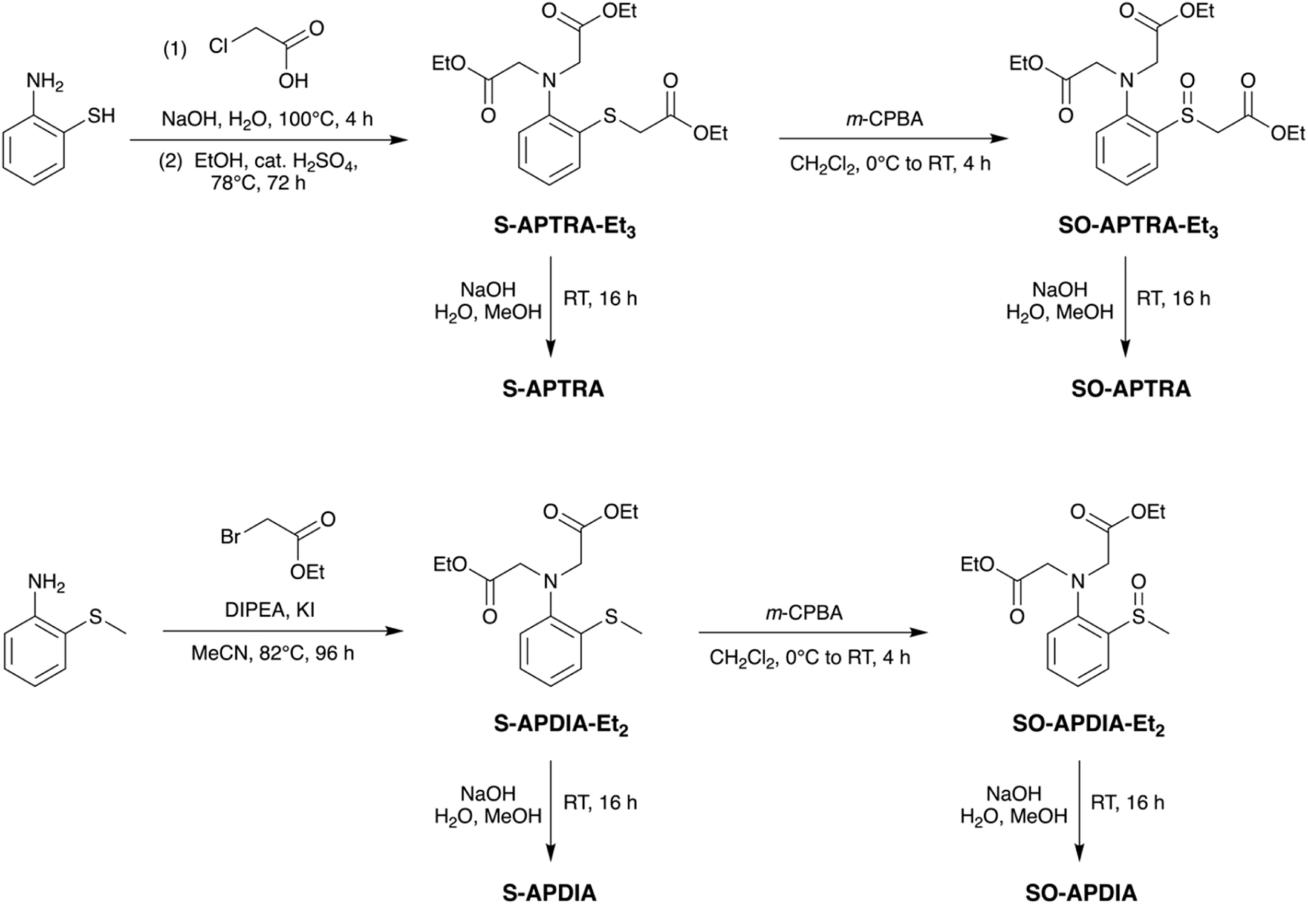
Synthetic Route to S-APTRA and SO-APTRA Starting from 2-Aminothiophenol, and to S-APDIA and SO-APDIA from Methyl(2-aminophenyl)sulfane

The synthetic strategy to access the ligands relies on the installation of the carboxylate arms through nucleophilic substitution with an α-haloacetate. Initially, we attempted to alkylate 2-aminothiophenol with ethylbromoacetate, a well-established route to azacarboxylates in general. However, this method gave complicated reaction mixtures from which only a small amount of the desired triester could be isolated by chromatography. (All experimental details and characterization of new compounds are provided in the Experimental Section or Supporting Information). In contrast, alkylation with chloroacetic acid in water gave superior results (Scheme 1). The resulting triacid was not isolated at this stage but rather was esterified in ethanol under acid catalysis to give the triester S-APTRA-Et_3_, which was amenable to chromatographic purification. The subsequent oxidation of this compound to the sulfoxide SO-APTRA-Et_3_ was accomplished under mild conditions using *m*-chloroperbenzoic acid (*m*-CPBA). The two proligands were converted to the desired, pentadentate carboxylate ligands by saponification with aqueous NaOH at ambient temperature. Meanwhile, alkylation of the *S*-methyl derivative of 2-aminothiophenol§ with BrCH_2_CO_2_Et gave S-APDIA-Et_2_ in tolerable yield, from which SO-APDIA-Et_2_ was obtained by oxidation. The tetradentate ligands S-APDIA and SO-APDIA were duly obtained by saponification of these two proligands, as for their pentadentate analogues.

### Absorption Spectroscopy: UV–Visible Spectra of the Free Ligands

Absorption spectroscopy was used to probe the metal-binding properties of the new ligands, which allowed comparison with literature data for APTRA. The ligands themselves are not significantly fluorescent, but conjugation to (or incorporation into) a fluorescent reporter unit can be readily envisaged for future preparation of fluorescent derivatives.

**Figure 2:**
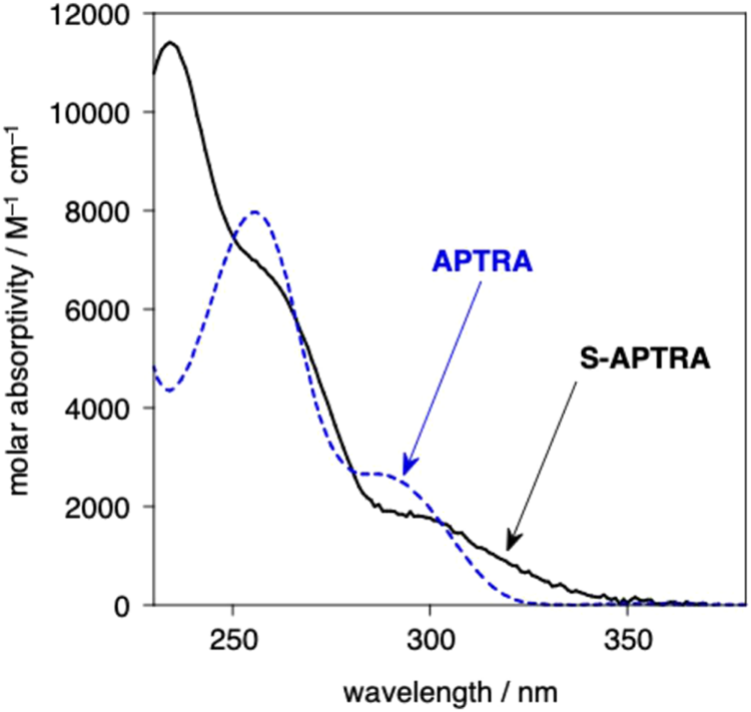
Absorption spectrum of S-APTRA in aqueous solution at pH 7.2 and 295 K (black solid line), together with a previously published spectrum of APTRA under comparable conditions.^20^

The UV–visible spectrum of the new ligand S-APTRA in aqueous solution at pH 7.2 shows three bands centered at about 235, 257, and 295 nm (Figure 2). The lowest-energy band of S-APTRA is red-shifted relative to that of APTRA (λ = 283 nm, Figure 2), and tails to longer wavelengths (around 350 and 320 nm, respectively). The difference reflects the higher energy filled *p* orbitals on sulfur raising the highest-occupied molecular orbitals, as typically found for the ^1^*L*_b_ band of thiophenols versus phenols, with a concomitant reduction in molar absorptivity at λ_max_.^23^

**Figure 3:**
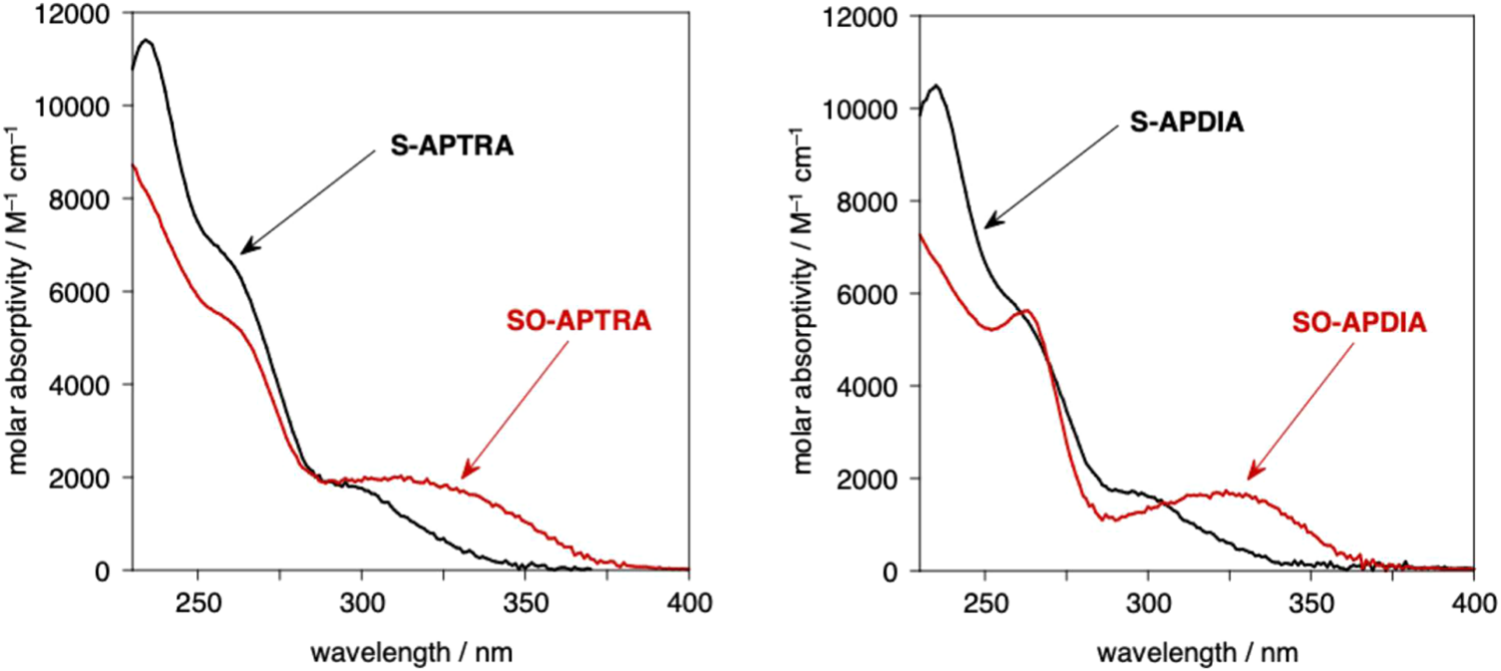
Left: absorption spectra of S-APTRA (black line) and SO-APTRA (red line) in aqueous solution at pH 7.2 and 295 K (black solid line). Right: corresponding spectra of S-APDIA and SO-APDIA under the same conditions.

The oxidation of the sulfide, S-APTRA, to the sulfoxide SO-APTRA leads to the appearance of a broad, lower energy band, λ_max_ ∼ 315 nm but tailing to >375 nm (Figure 3, left). The change can be understood in terms of the sulfoxide acting as an electron acceptor in an excited state that now has more donor–acceptor character (*cf*. the bathochromic shift of the benzene nucleus in aromatic sulfinic acids).^24^ The diacetate derivatives S-APDIA and SO-APDIA display very similar absorption spectra to their triacetate analogues (Figure 3, right), as would be expected, given that the “missing” acetate group should barely influence the electronic transitions associated with the aromatic ring. The low-energy band in SO-APDIA does, however, appear to be slightly more defined than in SO-APTRA, perhaps owing to the fewer possible conformers of the former.

### Absorption Spectroscopy: Effect of pH

**Figure 4:**
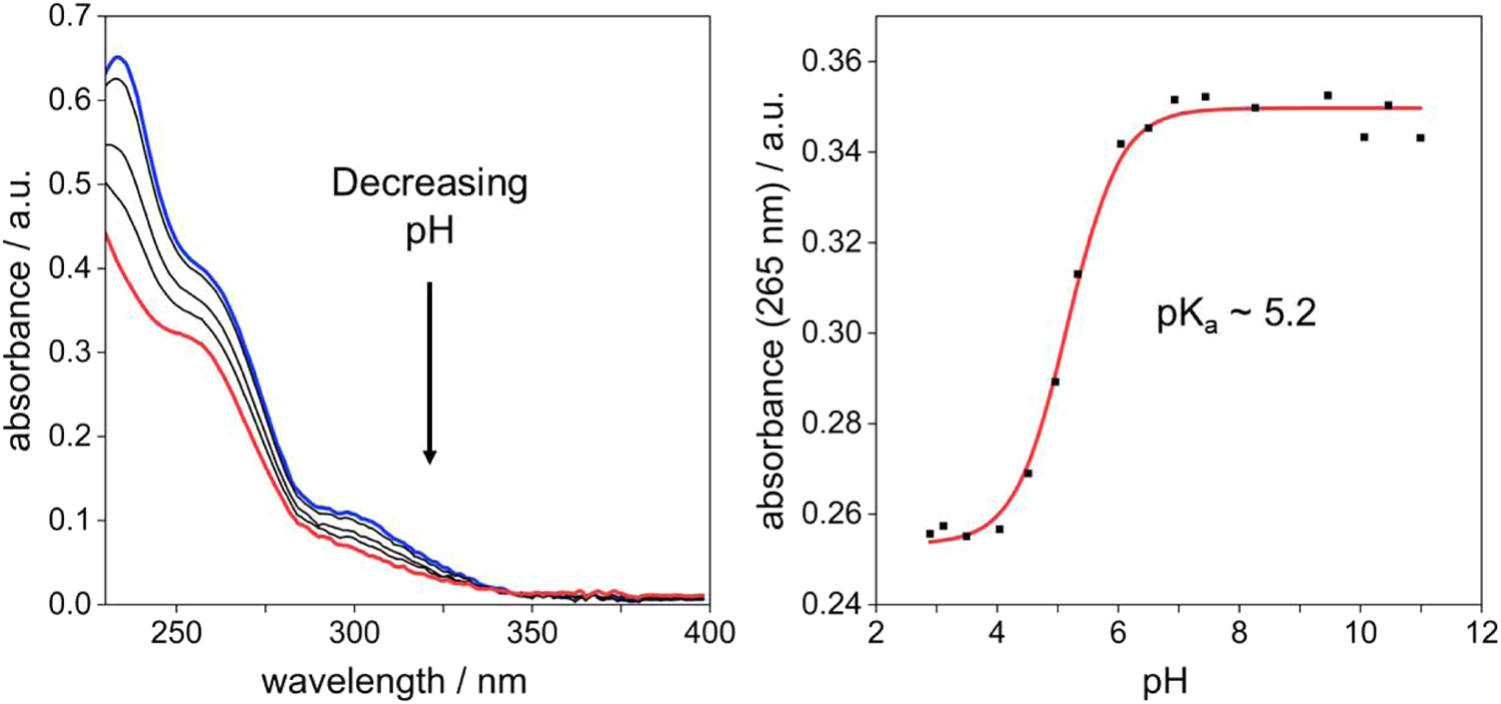
Left: the effect of pH on the absorption spectrum of S-APTRA (50 μM) in aqueous solution in the presence of KCl (0.1 M) at 295 K. The blue line refers to pH = 11 and the red to pH = 2, with black lines showing the spectra at intermediate pH values. Right: the absorbance at 265 nm plotted against pH, with the p*K*_a_ estimated from the fitted line (in red).

Prior to evaluating the effect of metal ions, it is important to determine the effect (if any) of pH changes on the absorption spectra, over the physiologically relevant range. Upon decreasing the pH < 7 using HCl, the intensity of absorption decreases panchromatically, but otherwise there are no pronounced changes or large shifts in the band positions, other than the low-energy band becoming less defined (Figure 4). This muted response contrasts with that of APTRA (and of APDAP, too), where both the profile and intensity of absorption change profoundly upon protonation.^21,25^ In each compound, the site of protonation is expected to be the nitrogen atom, and in APTRA and ADPAP, the nitrogen substituent no doubt dominates over the phenol oxygen in determining the frontier orbital energies. In contrast, in S-APTRA, the sulfur atom may have the dominant effect over the nitrogen, such that protonation of the latter leaves the frontier orbital energies relatively unchanged. From the change in intensity of absorption (λ = 265 nm was selected for monitoring), a ground-state p*K*_a_ of 5.2 was estimated for S-APTRA. This low value, in combination with the muted effect on the spectrum, evidently renders the compound promising for applications even in relatively acidic biological environments.

### Absorption Spectroscopy: Effect of Metal Ions

The response to the three main divalent metal ions of interest—Mg^2+^, Ca^2+^, and Zn^2+^—was investigated by titrating aqueous aliquots of the metal chloride salts into a HEPES-buffered aqueous solution of the ligand, in the presence of KCl. The addition of Mg^2+^ has no significant effect on the absorption spectrum of S-APTRA, even at quite high concentrations (Figure S1). That contrasts with the behavior of APTRA, whose absorption spectrum is substantially affected by Mg^2+^ (hence its use as a probe for this ion). The addition of Ca^2+^ results in a modest decrease in intensity of absorption across the spectrum, and a small blue shift of the bands. This hypsochromic shift (with no counterpart upon protonation) suggests that the Ca^2+^ ion may be associating—at least weakly—with the S lone pair. A dissociation constant *K*_d_ = 330 mM was estimated from the spectral changes (assuming 1:1 binding; Figure S2). This value is 2 orders of magnitude higher than that of APTRA, and much higher than that of cytosolic concentrations of Ca^2+^, indicating that S-APTRA binds Ca^2+^ so weakly that it should show no response to it in typical cellular environments. A screening of other divalent first row transition metal ions, typically much less bioavailable than Ca^2+^, was carried out under comparable conditions (Figure S3). Though there is inevitably some interference, the overall selectivity profile is on a par with the widely used ligands summarized in the introduction.

**Figure 5:**
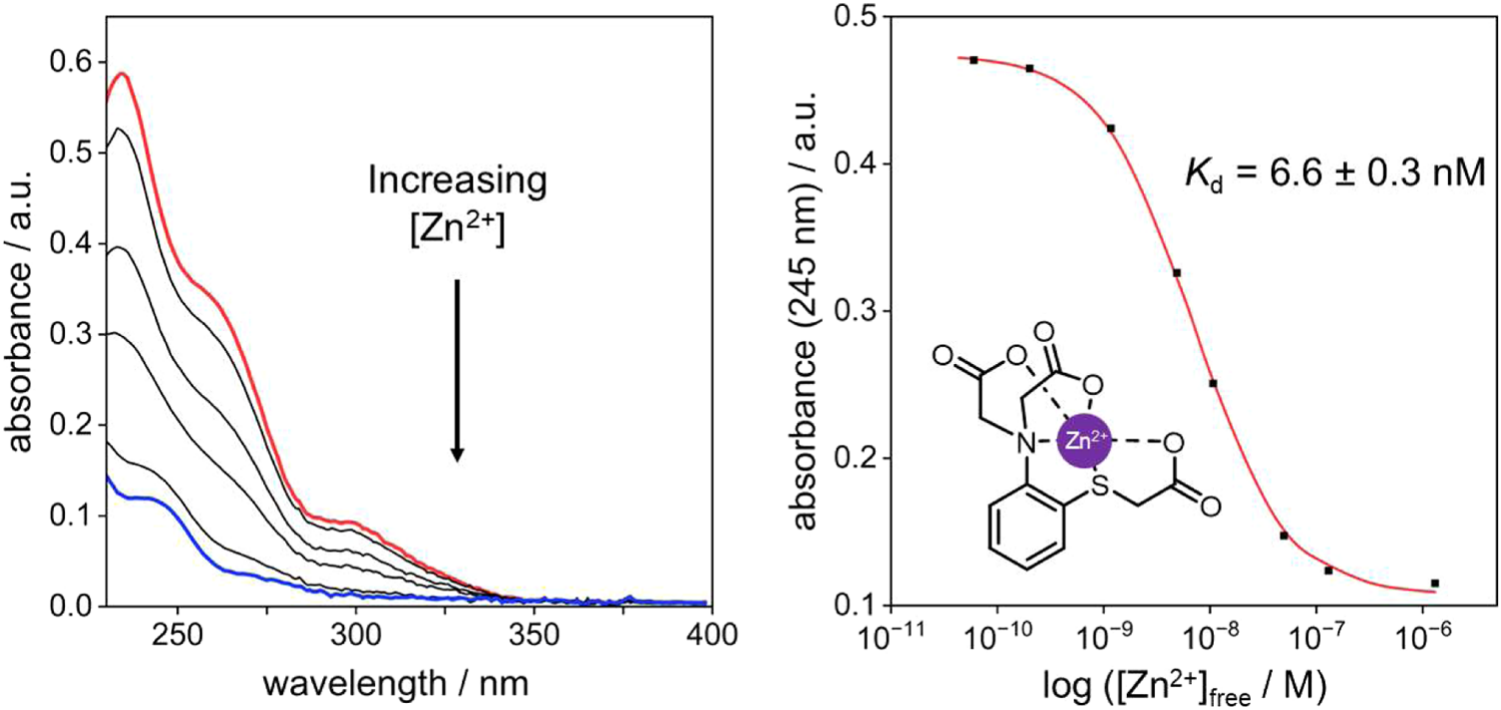
Effect of Zn^2+^ on the absorption spectrum of S-APTRA (50 μM) in buffered aqueous solution (50 mM HEPES, pH 7.2, 0.1 M KCl, 295 K). Left: the red and blue lines are the spectra in the absence of Zn^2+^ and in the presence of 1 μM Zn^2+^, respectively, with black lines at selected intermediate concentrations. Right: the absorbance at 245 nm plotted as a function of [Zn^2+^] (black squares, logarithmic axis) and the corresponding fitted line (red) from which *K*_d_ was estimated. The assumed pentadentate binding geometry is illustrated.

The addition of Zn^2+^ leads to a dramatic change in the absorption spectrum, even at very low concentrations of the metal. The intensity of absorption is strongly decreased, the lowest-energy band is markedly suppressed, and there is a blue shift of the higher energy bands (Figure 5). These changes may be indicative of Zn^2+^ binding strongly through both the N and S lone pairs, consistent with the “softer” nature of Zn^2+^ compared to Ca^2+^ and Mg^2+^. The very strong binding necessitated the use of a competitive binding experiment to quantify the affinity, from which a *K*_d_ of 6.6 ± 0.3 nM was estimated.¶ A 1:1 binding model was used, justified by evidence of 1:1 binding from a Job plot (“method of continuous variation”, Figure S4) and the absence of any other species in a ^1^H NMR titration with Zn^2+^ (*vide infra*). This value is somewhat lower than that of APTRA by a factor of about 2. The affinity for Zn^2+^ is sufficiently high to bind this metal ion at physiologically relevant concentrations, and is comparable to that of the commercial, widely adopted probe FluoZin-3 for instance^26^ (for which *K*_d_ = 15 nM). Moreover, it comes without any significant binding of—and hence interference from—Mg^2+^ or Ca^2+^ at their prevailing concentrations. We conclude that the substitution of APTRA’s phenolic oxygen by sulfur leads to a ligand, S-APTRA, with a binding profile well-suited to chelating biologically relevant amounts of Zn^2+^ selectively in aqueous media. The comparative data are summarized in Table 1.

**Table 1:** Comparison of the p*K*_a_ and Metal Binding Affinities of S-APTRA and APTRA,^a^^,^^b^ Evaluated by Absorption Spectroscopy in Aqueous Solution at 295 K^c^

	S-APTRA^d^	APTRA
p*K*_a_	5.2	5.5
*K*_d_ Mg^2+^	e	1.8 ± 0.1 mM
*K*_d_ Ca^2+^	326 ± 7 μM	9.8 ± 0.7 μM
*K*_d_ (Zn^2+^)	6.6 ± 0.3 nM	14 ± 2 nM
*K* Mg^2+^/M^–1^	e	560 ± 30
*K* Ca^2+^/M^–1^	(3.07 ± 0.06) × 10^3^	(1.02 ± 0.07) × 10^5^
*K* Zn^2+^/M^–1^	(1.53 ± 0.06) × 10^8^	(7 ± 1) × 10^7^

a p*K*_a_ value of APTRA is from ref (25).

b Metal-binding association constants *K* for APTRA are taken from ref (20).

c Dissociation constants *K*_d_ are given in the indicated units with the estimated uncertainty in this work from 3 measurements; corresponding association constants *K* are all given in M^–1^.

d Error values indicate the standard error of the mean for three titrations.

e Not determined: there is no significant response of S-APTRA to Mg^2+^.

### ^1^H NMR Spectroscopy: Effect of Zn^2+^ Binding

**Figure 6:**
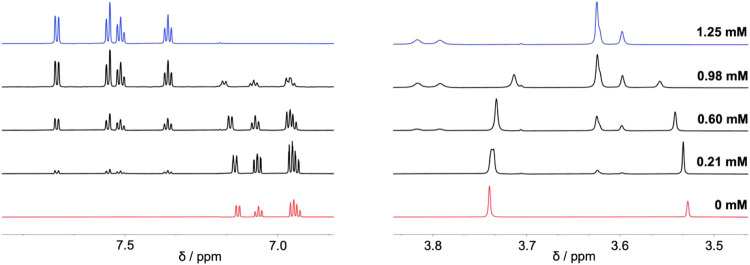
Aromatic and aliphatic regions of the ^1^H NMR spectrum of S-APTRA (1.14 mM) in D_2_O in the presence of Zn^2+^ at the concentrations indicated. The sulfate salt was used. The full-range spectra are shown in Figure S5.

The ^1^H NMR spectrum of S-APTRA shows three distinct aromatic signals alongside two singlets in the aliphatic region corresponding to the carboxylate side arms. Addition of Zn^2+^ to S-APTRA in D_2_O results in a new set of signals downfield from those of the ligand, suggesting the formation of a Zn^2+^ complex that is in equilibrium with S-APTRA in a slow-exchange regime (Figures 6 and S5). The aliphatic signals in the Zn^2+^ complex show additional splitting, due to the formation of a point chiral center at the S atom upon metal binding, resulting in protons such as those in the carboxylate side arms connected to the aniline nitrogen atom becoming diastereotopic.

The absence of any other set of signals that might have been attributed to an ML_2_ complex, for instance, together with the observation that saturation occurs at equimolar Zn^2+^ and S-APTRA, supports a 1:1 ML complex as the dominant binding mode, in accordance with the Job plot analysis. The slow exchange between the free ligand and complex is also consistent with the strong binding of S-APTRA to Zn^2+^ observed by absorption spectroscopy.

### Tuning Binding Strength through Denticity and/or Sulfur Oxidation State

**Figure 7:**
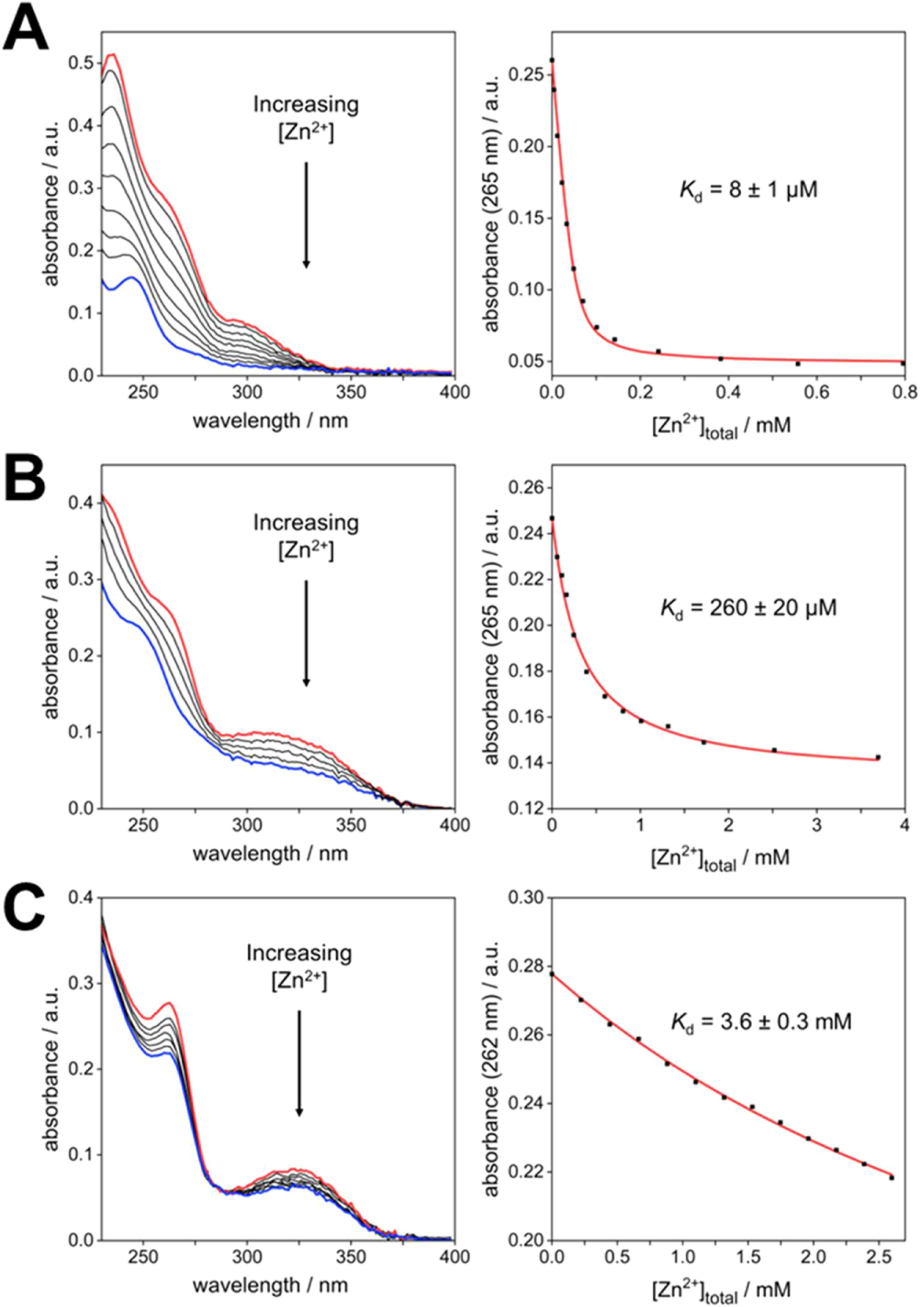
Left: The effect of Zn^2+^ on the absorption spectrum of (A) S-APDIA, (B) SO-APTRA, and (C) SO-APDIA, at a concentration of 50 μM in buffered aqueous solution (50 mM HEPES, 0.1 M KCl, 295 K) in each case. The red lines are the spectra in the absence of Zn^2+^ in each case, and the blue lines in the presence of (A) 0.8 mM ZnCl_2_, (B) 3.7 mM ZnSO_4_, and (C) 2.6 mM ZnSO_4_. (The sulfate was used in place of the chloride, as higher concentrations can be attained.) Right: The absorbance at a fixed wavelength (indicated in the *y*-axis), plotted as a function of concentration for each respective ligand and the corresponding fitted line.

When the tetradentate S-APDIA ligand is treated with Zn^2+^, an optical response resembling that of S-APTRA is observed, in the sense that there is a large decrease in the intensity of absorption and a significant blue shift (Figure 7A). Recalling that the absorption spectra of the two ligands are essentially identical (Figure 3), the interaction of the metal ion with the S and N lone pairs is expected to elicit similar changes within the aminothiophenol unit {a 1:1 binding model was again supported by a Job plot (Figure S8) and literature precedent for a tetradentate APTRA analogue^14b^}. Not surprisingly, the lower denticity of S-APDIA results in weaker binding: a *K*_d_ value of around 8 μM was estimated from the data, around 10^3^ higher than for S-APTRA. The weaker binding is evidently a manifestation of the well-known, largely entropically driven chelate effect. This simple structural modification thus renders S-APDIA potentially suited for probing Zn^2+^ in zinc-rich samples. There is no response to Mg^2+^ or Ca^2+^ (Figures S6 and S7 respectively).

The oxidation of the sulfide to the sulfoxide substantially reduces the affinity of the ligands toward Zn^2+^. For SO-APTRA, there is still a hypsochromic shift in the band at 265 nm and a modest increase in the intensity of absorption across the spectrum (Figure 7B), but the *K*_d_ value increases to around 260 μM; i.e., the affinity for Zn^2+^ is reduced by 4–5 orders of magnitude compared to S-APTRA. In SO-APDIA, the combination of the change to sulfoxide *and* the lower denticity further suppresses binding, leading to a *K*_d_ value of around 3.6 mM, with small concomitant changes in the spectrum, and no shift in the band at 265 nm (Figure 7C). The contrasting results for the four ligands with Zn^2+^ are summarized in Table 2. The suppression of zinc binding upon sulfur oxidation may be understood by considering the two possible modes by which the >S=O unit may participate in the binding of the metal ion: either through the sulfur or the oxygen. In the former case, a lower binding affinity would be anticipated through the inductive effect of the oxygen atom. In the latter, the binding of the metal ion to the sulfoxide oxygen atom would create two six-membered (as opposed to five-membered) chelate rings within the complex. Except for small metal ions, the formation of five-membered chelates is usually thermodynamically favored over six-membered rings.^27^

**Table 2:** Summary of the *K*_d_ Values for the Four New Ligands with Zn^2+^ in Aqueous Solution^a^

	***K***_**d**_**Zn**^**2+**^
S-APTRA	6.6 ± 0.3 nM
S-APDIA	8 ± 1 μM
SO-APTRA	260 ± 20 μM
SO-APDIA	3.6 ± 0.3 mM

a Error values indicate the standard error of the mean for three titrations.

## Concluding Remarks

The aminothiophenol-based ligands described here show much promise for elaboration into a new family of zinc-selective sensors. The S-APTRA ligand binds Zn^2+^ in the nM range and with high selectivity over Ca^2+^ and Mg^2+^ at their biologically relevant concentrations. The high denticity of the ligand and the net negative charge on the complex render it attractive for the development of probes that will not suffer from ternary complex formation.^18^ Moreover, it is a carboxylate-based ligand (in contrast to, say, the DPA systems) and could thus benefit from advantages offered by carboxylates, such as improved water solubility, and the possibility to convert them to acetoxymethyl esters for enhanced cell permeability and retention within the cell following ester hydrolysis. The binding affinity can be tuned through decreasing denticity, with the diacetate derivative S-APDIA binding Zn^2+^ in the μM range, making it potentially suited for probing the higher end of biologically relevant concentrations. Meanwhile, oxidation at sulfur essentially prevents binding to Zn^2+^ at the typical concentrations found *in vivo*, which raises the intriguing possibility of a dual-modality sensory mechanism for reactive oxygen species *and* zinc. At this proof-of-concept stage, the detection strategy for Zn^2+^ is evidently limited to UV absorption, but the future conjugation or incorporation of these ligating units into suitable fluorophores should lead to practicable sensors amenable to fluorescence microscopy techniques.

## Experimental Section

Details of general synthetic and spectroscopic methods are given in the Supporting Information, together with the synthesis of known intermediates. The synthesis and characterization of the new compounds are described below, complemented by ^1^H and ^13^C NMR spectra in the Supporting Information.

### S-APTRA-Et_3_

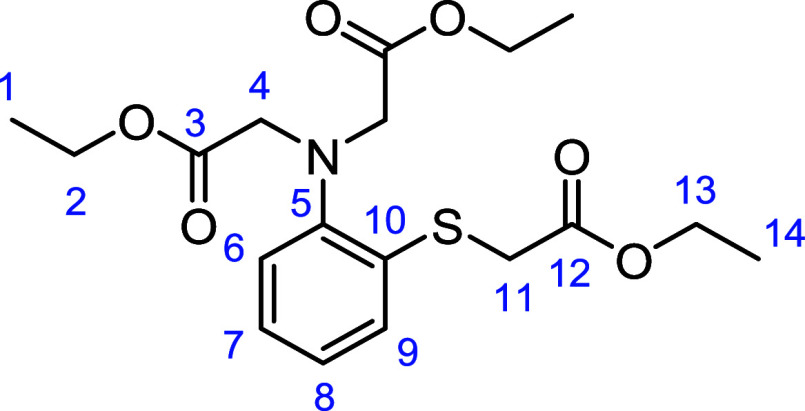



2-Aminothiophenol (1.0 mL, 10 mmol) and chloroacetic acid (4.73 g, 50.0 mmol, 5 equiv) were added to a two-necked 100 mL round-bottomed flask. Aqueous NaOH (10 mL, 7 M, 50 mmol, 5 equiv) was added. The gray suspension was heated to reflux and the pH was monitored every 15 min for 4 h, with solid portions of NaOH being added if the pH dropped <10. The crude mixture was transferred to a round-bottomed flask and the solvent was removed under reduced pressure. Ethanol (35 mL) and conc. sulfuric acid (4.2 mL) were added, and the solution was refluxed for 72 h. The solvent was removed under reduced pressure; ethyl acetate (30 mL) was added, and the resulting suspension was filtered and washed with 10% NaOH (1 × 20 mL) and brine (2 × 20 mL). The organic layer was dried over MgSO_4_, filtered, and the solvent removed under reduced pressure to give a gray residue. Purification by silica gel chromatography (100% hexane to 50% hexane/50% ethyl acetate) followed by reverse-phase silica gel chromatography (10% MeCN/90% water to 100% MeCN) gave S-APTRA-Et_3_ as a yellow oil (0.37 g, 10%). *R*_f_: 0.57 (silica, 30% ethyl acetate, 70% hexane). ^1^H NMR (599 MHz, CDCl_3_): δ_H_ 7.33 (1H, dd, *J* 8.0, 1.5, H^9^), 7.21 (1H, dd, *J* 8.0, 1.4, H^6^), 7.20–7.14 (1H, m, H^7^), 7.04–6.98 (1H, m, H^8^), 4.18 (4H, s, H^4^), 4.15–4.07 (6H, m, H^2^ and H^13^), 3.75 (2H, s, H^11^), 1.21 (6H, t, *J* 7.0 H^1^), 1.17 (3H, t, *J* 7.0, H^14^). ^13^C NMR (151 MHz, CDCl_3_): δ_c_ 170.9 (C^3^), 170.1 (C^12^), 149.1 (C^5^), 131.4 (C^9^), 130.2 (C^10^), 127.7 (C^7^), 124.6 (C^8^), 123.6 (C^6^), 61.4 (C^13^), 60.8 (C^2^), 53.9 (C^4^), 34.7 (C^11^), 14.3 (C^1^), 14.2 (C^14^). LC/ESI-LRMS (CH_3_CN/H_2_O, 0.1% FA) *t*_R_ = 2.85 min; (+) *m*/*z* 384 [M + H]^+^, 406 [M + Na]^+^; ESI-HRMS (+) 384.1481 *m*/*z* [M + H]^+^, calculated for [C_18_H_26_NO_6_S]^+^ 384.1482.

### S-APTRA

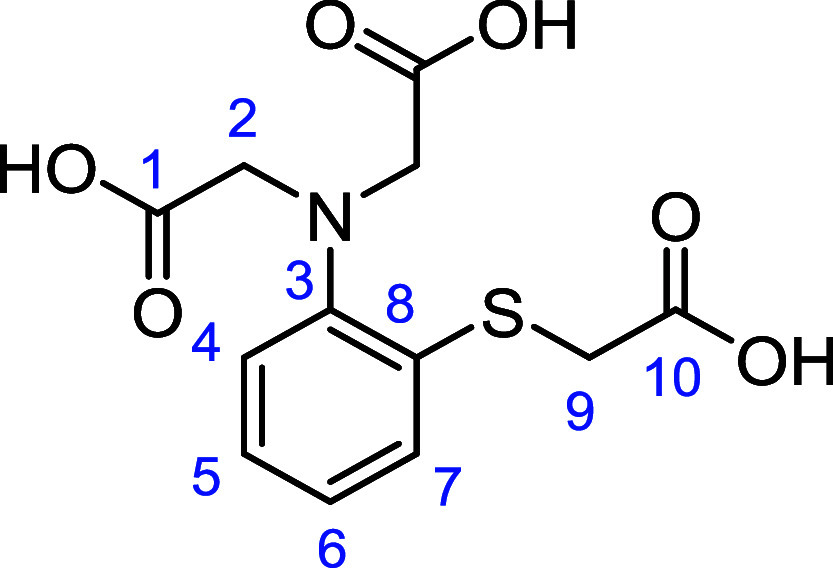



A glass vial containing S-APTRA-Et_3_ (21 mg, 0.055 mmol) was charged with MeOH (1 mL) and purged with N_2_. Aqueous NaOH (1.0 M, 0.22 mL, 0.22 mmol) was added and the pale green solution was stirred at room temperature for 16 h. The solution was then diluted with water (2 mL) and the pH adjusted to 7 using 1 M HCl. The solvent was removed under reduced pressure to give a white solid (quant. yield). ^1^H NMR (599 MHz, D_2_O, pD 7): δ_H_ 7.25 (1H, dd, *J* 7.8, 1.5, H^7^), 7.20–7.16 (1H, m, H^5^), 7.09–7.03 (2H, m, H^4^ and H^6^), 3.81 (4H, s, H^2^), 3.67 (2H, s, H^9^). ^13^C NMR (151 MHz, D_2_O, pD 7): δ_C_ 178.9 (C^1^), 177.3 (C^10^), 148.5 (C^3^), 130.0 (C^8^), 127.4 (C^7^), 125.9 (C^5^), 123.2 (C^4^), 119.3 (C^6^), 57.3 (C^2^), 37.6 (C^9^). LC/ESI-LRMS (CH_3_CN/H_2_O, 0.1% FA) *t*_R_ = 1.7 min; (+) *m*/*z* 300 [M + H]^+^, 322 [M + Na]^+^; ESI-HRMS (+) 300.0515 *m*/*z* [M + H]^+^, calculated for [C_12_H_14_NO_6_S]^+^ 300.0542.

### SO-APTRA-Et_3_

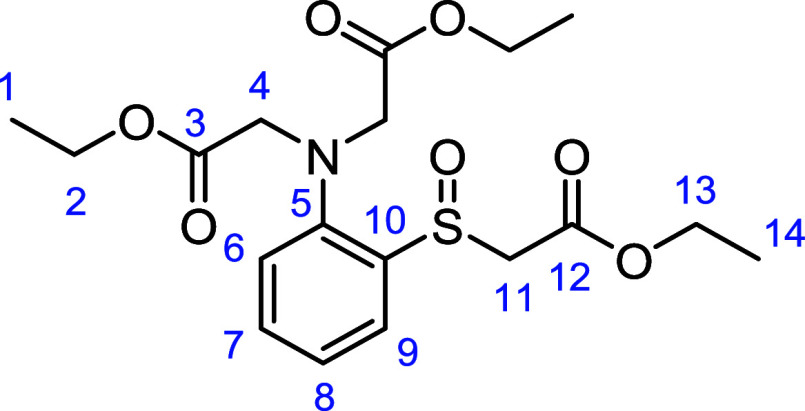



To an oven-dried Schlenk flask was added a solution of S-APTRA-Et_3_ (222 mg, 0.578 mmol) in dry CH_2_Cl_2_ (2.5 mL) under N_2_. The solution was cooled to 0 °C and a solution of 70–75% *m*-CPBA (133 mg, 0.578 mmol) was added dropwise over 15 min. The reaction was stirred for 1 h at 0 °C under N_2_. The pale-green solution was diluted with CH_2_Cl_2_ (10 mL) and washed with saturated NaHCO_3_ solution (2 × 10 mL) and brine (1 × 10 mL). The organic layer was dried over MgSO_4_, filtered, and the solvent was removed under reduced pressure to give a colorless oil. Purification by column chromatography (100% hexane to 100% ethyl acetate) gave SO-APTRA-Et_3_ as a colorless oil (184 mg, 80%). *R*_f_: 0.35 (silica, hexane to 50% hexane/ethyl acetate). ^1^H NMR (599 MHz, CDCl_3_) δ_H_ 7.91 (1H, dd, *J* 8.0, 1.6, H^9^), 7.45–7.41 (1H, m, H^7^), 7.35–7.31 (1H, m, H^8^), 7.28 (1H, dd, *J* 7.9, 1.1, H^6^), 4.54 (1H, d, *J* 14.0, H^11A^), 4.20 (2H, d, *J* 18.0, H^4A^), 4.17–4.09 (6H, m, H^2^ and H^13^), 3.95 (2H, d, *J* 18.0, H^4B^), 3.78 (1H, d, *J* 14.0, H^11B^), 1.22 (9H, m, H^1^ and H^14^). The diastereotopicity of H^4^ and H^11^ gives rise to AB spin systems for these nuclei. ^13^C NMR (151 MHz, CDCl_3_): δ_C_ 170.1 (C^3^), 165.9 (C^12^), 146.6 (C^5^), 137.2 (C^10^), 131.9 (C^7^), 125.6 (C^8^), 125.5 (C^9^), 123.0 (C^6^), 61.9 (C^13^), 61.2 (C^2^), 58.1 (C^11^), 54.8 (C^4^), 14.3 (C^1^), 14.2 (C^14^). LC/ESI-LRMS (CH_3_CN/H_2_O, 0.1% FA) *t*_R_ = 2.3 min; (+) *m*/*z* 400 [M + H]^+^, 423 [M + Na]^+^; ESI-HRMS (+) 400.1430 *m*/*z* [M + H]^+^, calculated for [C_28_H_26_NO_7_S]^+^ 400.1407.

### SO-APTRA

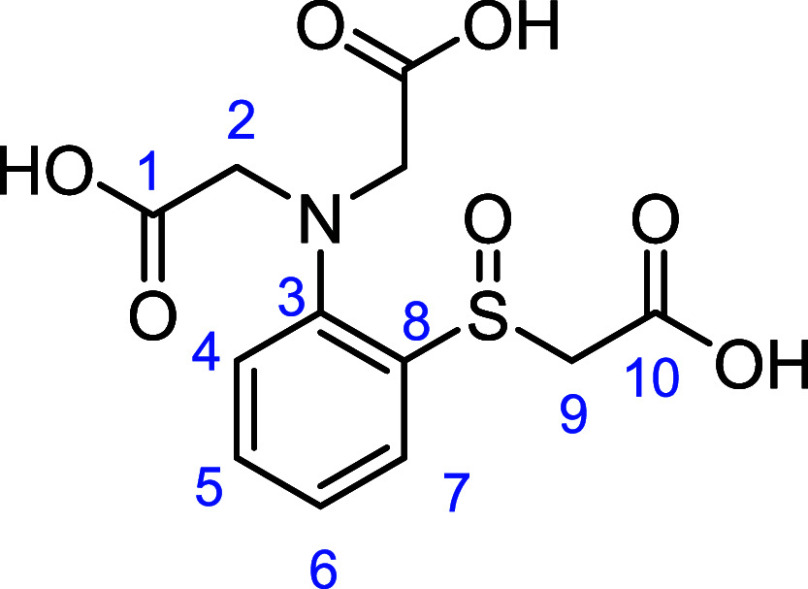



A glass vial containing SO-APTRA-Et_3_ (21 mg, 0.053 mmol) was charged with MeOH (1 mL) and purged with N_2_. NaOH solution (1.0 M, 0.21 mL, 0.21 mmol) was added and the pale green solution was stirred at room temperature for 16 h. The solution was then diluted with water (2 mL) and the pH adjusted to 7 using 1 M HCl. The solvent was removed under reduced pressure to give a white solid (quant. yield). ^1^H NMR (599 MHz, D_2_O): δ_H_ 7.82 (1H, dd, *J* 8.0, 1.5, H^7^), 7.60–7.51 (1H, m, H^5^), 7.33–7.27 (1H, m, H^6^), 7.17 (1H, d, *J* 8.5, H^4^), 4.58 (1H, d, *J* 15.5, H^9A^), 3.98 (2H, d, *J* 17.5, H^2A^), 3.85 (2H, d, *J* 17.5, H^9B^), 3.57 (1H, d, *J* 15.5, H^9B^). The diastereotopicity of H^9^ gives rise to an AB spin system for this nucleus. ^13^C NMR (151 MHz, D_2_O): δ_C_ 178.2 (C^1^), 172.1 (C^10^), 148.6 (C^3^), 132.3 (C^5^), 131.6 (C^8^), 125.0 (C^7^), 123.0 (C^6^), 120.2 (C^4^), 61.3 (C^9^), 58.2 (C^2^). LC/ESI-LRMS (CH_3_CN/H_2_O, 0.1% FA) *t*_R_ = 1.24 min; (+) *m*/*z* 316 [M + H]^+^, 338 [M + Na]^+^; ESI-HRMS (+) 316.0482 *m*/*z* [M + H]^+^, calculated for C_12_H_14_NO_7_S 316.0491.

### S-APDIA-Et_2_

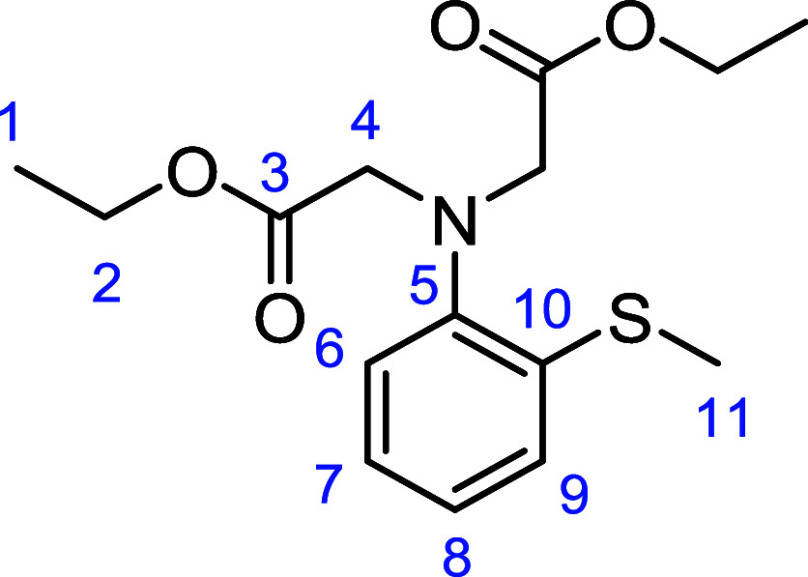



Ethyl bromoacetate (1.1 mL, 11 mmol, 3 equiv) and *N*,*N*-diisopropylethylamine (3.2 mL, 18 mmol, 5 equiv) were added to a solution of methyl(2-aminophenyl)sulfane (490 mg, 3.63 mmol) and potassium iodide (1.21 g, 7.26 mmol, 2 equiv) in dry acetonitrile (6 mL) in an oven-dried Schlenk tube under N_2_. The resulting brown suspension was heated at reflux under N_2_ for 48 h, at which point a further portion of ethyl bromoacetate (0.4 mL, 4 mmol, 1.1 equiv) and *N,N*-diisopropylethylamine (1.0 mL, 5.7 mmol, 1.6 equiv) was added. The solution was refluxed for a further 48 h until the sulfide starting material had been consumed (monitored by LCMS). The solution was diluted in CH_2_Cl_2_ (30 mL) and poured into a separating funnel containing water. The layers were separated, and the aqueous layer was extracted further with CH_2_Cl_2_ (2 × 30 mL). The organic extracts were combined, dried over MgSO_4_, and filtered. The solvent was removed under reduced pressure, redissolved in CH_2_Cl_2_ (20 mL), and adsorbed onto silica. Purification by silica gel chromatography (hexane to ethyl acetate) gave the title compound as a brown oil (177 mg, 16%). ^1^H NMR (599 MHz, CDCl_3_): δ_H_ 7.26–7.24 (1H, m, H^9^), 7.13–7.09 (1H, m, H^7^), 7.07–7.04 (2H, m, H^6^ and H^8^), 4.15–4.05 (8H, m, H^2^ and H^4^), 2.40 (3H, s, H^11^), 1.20 (6H, t, *J* 7.2, H^1^). ^13^C NMR (151 MHz, CDCl_3_): δ_C_ 170.8 (C^3^), 146.8 (C^5^), 134.9 (C^10^), 125.6 (C^7^), 125.0 (C^6^ or C^8^), 124.9 (C^6^ or C^8^), 123.6 (C^9^), 60.5 (C^2^), 53.6 (C^4^), 14.8 (C^11^), 14.1 (C^1^). LC/ESI-LRMS (CH_3_CN/H_2_O, 0.1% FA) *t*_R_ = 3.0 min; (+) *m*/*z* 312 [M + H]^+^, 335 [M + Na]^+^; ESI-HRMS (+) 312.1274 *m*/*z* [M + H]^+^, calculated for [C_15_H_22_NO_4_S]^+^ 312.1270.

### S-APDIA

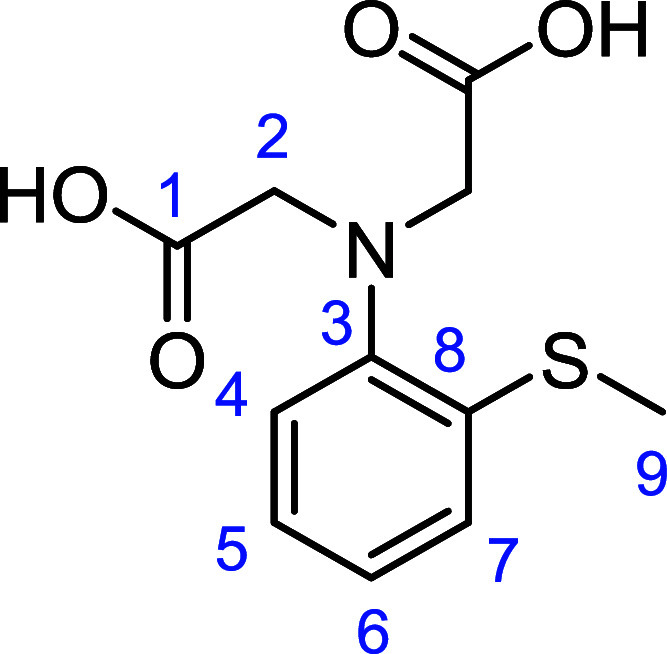



A glass vial containing S-APDIA-Et_2_ (10 mg, 0.034 mmol) dissolved in MeOH (0.1 mL) was purged with N_2_. Aqueous NaOH (1 M, 0.1 mL, 0.1 mmol) was added and the pale-yellow solution was stirred at room temperature for 1 h. The solution was then diluted with water (10 mL) and the pH adjusted to 7 using 1 M HCl. The solvent was removed under reduced pressure to give a white solid (quant. yield). ^1^H NMR (599 MHz, D_2_O, pD 7): δ_H_ 7.51 (1H, d, *J* 9.5, H^7^), 7.40–7.22 (3H, m, H^4^, H^5^ and H^6^), 3.95 (2H, s, H^2^), 2.61 (1H, s, H^9^). ^13^C NMR (151 MHz, D_2_O): δ_C_ 177.6 (C^1^), 147.2 (C^3^), 131.7 (C^8^), 127.7 (C^7^), 126.2 (C^4^, C^5^ or C^6^), 124.9 (C^4^, C^5^ or C^6^), 119.7 (C^4^, C^5^ or C^6^), 57.9 (C^2^), 15.4 (C^9^). LC/ESI-LRMS: (CH_3_CN/H_2_O, 0.1% FA) *t*_R_ = 1.5 min; (+) *m*/*z* 255 [M + H]^+^; ESI-HRMS: (+) 256.0637 *m*/*z* [M + H]^+^, calculated for [C_11_H_14_NO_4_S]^+^ 256.0642.

### SO-APDIA-Et_2_

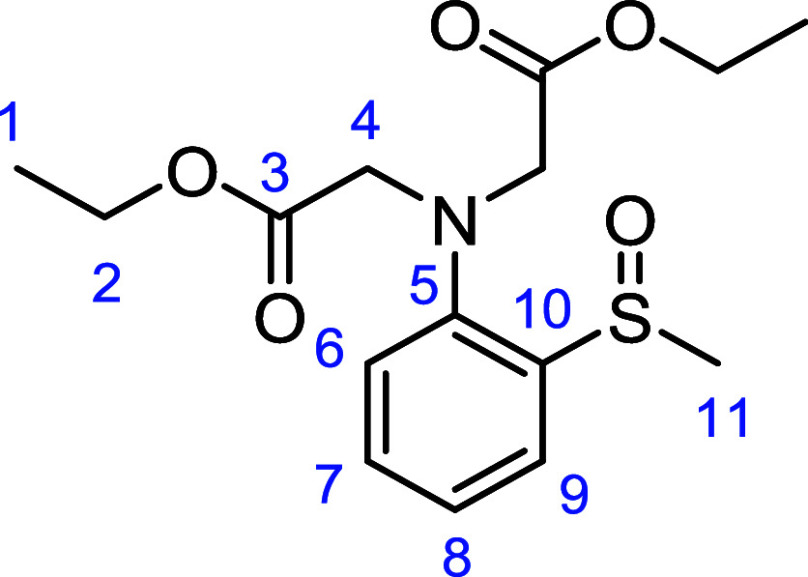



To an oven-dried Schlenk was added a solution of S-APDIA-Et_2_ (177 mg, 0.57 mmol) in CH_2_Cl_2_ (2.5 mL) under N_2_. The solution was cooled to 0 °C and a solution of 70–75% *m*-CPBA (131 mg, 0.57 mmol) in CH_2_Cl_2_ (5 mL) was added dropwise over 15 min. The reaction was stirred for 1 h at 0 °C. The pale green solution was diluted with CH_2_Cl_2_ (10 mL) and washed with saturated NaHCO_3_ solution (2 × 10 mL) and brine (1 × 10 mL). The organic layer was dried over MgSO_4_, filtered, and the solvent was removed under reduced pressure to give a colorless oil. Purification by column chromatography (100% hexane to 100% ethyl acetate) gave SO-APDIA-Et_2_ as a colorless oil (184 mg, 80%). *R*_f_: 0.3 (silica, hexane to 50% hexane/ethyl acetate). ^1^H NMR: (599 MHz, CDCl_3_): δ_H_ 7.92 (1H, dd, *J* 8.0, 1.5, H^9^), 7.43–7.37 (1H, m, H^7^), 7.34–7.29 (1H, m, H^8^), 7.25–7.23 (1H, m, H^6^), 4.17 (2H, d, *J* 18.0, H^4A^), 4.14–4.08 (4H, m, H^2^), 3.93 (2H, d, *J* 18.0, H^4B^), 2.91 (3H, s, H^11^), 1.22 (6H, t, *J* 7.0, H^1^). ^13^C NMR (151 MHz, CDCl_3_): δ_C_ 170.0 (C^3^), 146.2 (C^5^), 140.1 (C^10^), 131.3 (C^7^), 125.5 (C^8^), 124.3 (C^9^), 122.6 (C^6^), 61.0 (C^2^), 54.6 (C^4^), 41.8 (C^11^), 14.1 (C^1^). LC/ESI-LRMS (CH_3_CN/H_2_O, 0.1% FA) *t*_R_ = 2.1 min; (+) *m*/*z* 328 [M + H]^+^, 350 [M + Na]^+^; ESI-HRMS (+) 328.1213 *m*/*z* [M + H]^+^, calculated for [C_15_H_22_NO_5_S]^+^ 328.1219.

### SO-APDIA

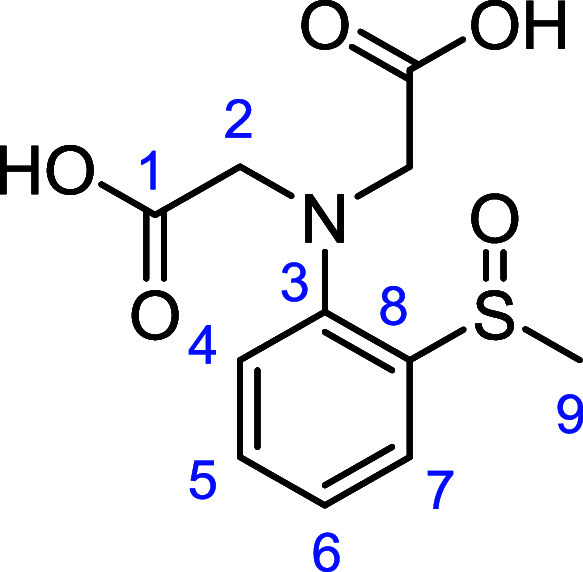



A glass vial containing SO-APDIA-Et_2_ (13 mg, 0.0158 mmol) was charged with MeOH (0.1 mL) and purged with N_2_. Aqueous NaOH (1 M, 0.1 mL, 0.1 mmol) was added and the pale green solution was stirred at room temperature for 1 h. The solution was then diluted with water (10 mL) and the pH adjusted to 7 using 1 M HCl. The solvent was removed under reduced pressure to give a white solid (quant. yield). ^1^H NMR (599 MHz, D_2_O, pD 7): δ_H_ 8.02–7.93 (1H, m, H^7^), 7.73–7.62 (1H, m, H^5^), 7.49–7.36 (1H, m, H^6^), 7.30–7.13 (1H, d, *J* 8.5, H^4^), 4.02–3.97 (4H, s, H^2^), 3.12–3.07 (3H, s, H^9^). ^13^C NMR: (151 MHz, D_2_O): δ_C_ 178.5 (C^1^), 148.9 (C^3^), 132.9 (C^4^, C^5^, C^6^ or C^7^), 124.8 (C^4^, C^5^, C^6^ or C^7^), 123.0 (C^4^, C^5^, C^6^ or C^7^), 119.6 (C^4^, C^5^, C^6^ or C^7^), 58.4 (C^2^), 40.9 (C^9^). LC/ESI-LRMS (CH_3_CN/H_2_O, 0.1% FA) *t*_R_ = 1.5 min; (+) *m*/*z* 256 [M + H]^+^; ESI-HRMS (+) 272.0609 *m*/*z* [M + H]^+^, calculated for [C_11_H_14_NO_5_S]^+^ 272.0593. C^8^ was not observed in the ^13^C NMR spectrum.

## Data Availability

The data supporting this article, including NMR spectra of all new materials, have been included as part of the Supporting Information.

## Supporting Information Available

Details of general synthetic and spectroscopic methods; synthesis and characterization of intermediates; absorption spectra of S-APTRA and S-APDIA in the presence of Mg^2+^ and Ca^2+^ (PDF)

## Footnotes

‡ “Free zinc” in this context refers to the hydrated cation, with no other endogenous ligands coordinated.

§ i.e., methyl(2-aminophenyl)sulfane, which was prepared from bis(2-nitrophenyl)disulfide by successive reduction of the disulfide to the thiol, *S*-methylation, and reduction of the nitro group to the amine. Full details are provided in the Supporting Information.

¶ The titration with Zn^2+^ was carried out in the presence of ethylene glycol-bis(2-aminoethyl ether)tetraacetate (EGTA): details of the procedure are provided in the Supporting Information.
