# Supplementary material for: Enhanced Binding of Zn2+ Using a Sulfur Version of o-Aminophenol-Triacetate (APTRA): Introducing S-APTRA and Derivatives
Source: Inorg Chem. 2025 May 7;64(19):9509–18. doi: 10.1021/acs.inorgchem.5c00275 (PMC12093301; doi:10.1021/acs.inorgchem.5c00275)
Supplement: Supplementary file 1 — ic5c00275_si_001.pdf [file ic5c00275_si_001.pdf]

SUPPORTING INFORMATION

Enhanced binding of Zn^{2+} using a sulfur version of *o*-aminophenol-triacetate (APTRA): introducing S-APTRA and derivatives

Christopher Hogg, Laura L. Duncan, David Parker and J. A. Gareth Williams*[a]

Department of Chemistry, Durham University, South Road, Durham, DH1 3LE, U.K.

** E-mail: j.a.g.williams@durham.ac.uk*

Contents

Experimental Methods S2
- Synthesis S2
- Optical Spectroscopy S2
- pH measurement and pK_a determination S2
- Metal Binding Studies and Binding Constant Determination S3
- ^{1}H NMR Titration S3

Synthetic Intermediates S4

Additional Binding Experiments S6
- S-APTRA with Mg^{2+} S6
- S-APTRA with Ca^{2+} S6
- Metal Competition Studies of S-APTRA S7
- Job Plot of S-APTRA with Zn^{2+} S7
- ^{1}H NMR Titration of S-APTRA with Zn^{2+} S8
- S-APDIA with Mg^{2+} S8
- S-APDIA with Ca^{2+} S9
- Job Plot of S-APDIA with Zn^{2+} S9

^{1}H and ^{13}C NMR spectra of all new compounds reported S11

References S19

Experimental Methods

Synthesis

All chemicals purchased from external suppliers were used as received, without further purification. All solvents used were of standard laboratory grade or higher; acetonitrile was dried by an SPS solvent system. Thin-layer chromatography was performed on silica (Merck Art 5554) or neutral alumina (Merck Art 5550) and visualised under UV irradiation (254 nm and 366 nm) or by staining with potassium permanganate or phosphomolybdic acid. Normal-phase column chromatography was performed manually with silica (Fluorochem Silica Gel, 230 – 400 mesh) or by using a Teledyne Combi-flash instrument equipped with RediSep R_f silica cartridge pre-packed columns.

The ^{1}H and ^{13}C NMR spectra of the compounds reported in the main text were acquired on a Varian 600 MHz instrument; assignments were made with the aid of two-dimensional spectra (COSY, NOESY, HSQC and HMBC). The spectra of the intermediate compounds below were recorded on Bruker Avance or Varian Mercury 400 NMR spectrometers. Electrospray ionization mass spectra (in positive and negative modes) were obtained on an SQD mass spectrometer interfaced with an Acquity UPLC system with acetonitrile as the carrier solvent. Spectra acquired using an atmospheric solids atomization probe were recorded on a Waters Xevo QToF mass spectrometer.

Optical Spectroscopy

UV-visible absorption spectra were measured using a double-beam Uvikon XS spectrometer operated with LabPower software. The sample was held in a quartz cuvette of pathlength 1 cm, with the pure solvent in an optically matched cuvette in the reference beam.

pH measurement and pK_a determination

The pH of solutions was recorded using a Jenway 3510 pH meter in combination with a Jenway 924 005 pH electrode. The pH probe was calibrated before each independent titration using commercial buffer solutions of pH 4.0, 7.0 and 10.0. Samples were prepared with a background of constant ionic strength (I = 0.1 M KCl, 298 K). Aqueous solutions were titrated to acid using aqueous HCl at 0.025 M, 0.05 M, or 0.1 M. The resulting sigmoidal curve of absorbance versus pH was fitted by a nonlinear least-squares iterative analysis in Microsoft Excel.

Metal Binding Studies and Binding Constant Determination

All divalent metal binding studies for the addition of Mg^{2+}, Ca^{2+} and Zn^{2+} were carried out in buffered solutions of 50 mM HEPES and 100 mM KCl maintained at pH 7.2. The chloride salts $MgCl_2$, $CaCl_2$ and $ZnCl_2$ were used in each instance, except for the titrations of SO-APTRA and SO-APDIA with Zn^{2+}. Here, $ZnSO_4$ was used instead, as it allows the higher necessary Zn^{2+} concentrations to be attained without the without unwanted precipitation that occurred when attempting to use $ZnCl_2$ in the mM range. Concentrations of the ligand used during the titration are stated in each figure caption. Stock solutions of metal ions were prepared to contain the same concentration of the ligand as in the cuvette, to avoid sample dilution over the course of the titration. Small aliquots of metal ion solutions were added in each instance, and the sample was left for 5 min after each addition, to ensure equilibration prior to recording the absorption spectrum. For K_d > 1 μM, a least-squares iterative fitting iterative was used to obtain the K_d value.[1,2] Titrations were repeated three times: the mean and standard error are reported in each case.

For K_d < 1 μM, a competitive binding study was carried out using ethylene glycol-bis(β-amino-ethyl ether)-*N*,*N*,*N′*,*N′*-tetraacetic acid (EGTA). Aliquots from a HEPES buffered solution (50 mM HEPES, 100 mM KCl) of $ZnCl_2$ or $Zn(SO_4)$ (~1 mM), EGTA (1 mM) and ligand (50 μM) were added to a solution of HEPES buffered solution (50 mM HEPES, 100 mM KCl) of EGTA (1 mM) and ligand (50 μM). The concentration of free zinc, $[Zn^{2+}]$, was calculated from the total $[Zn^{2+}]_t$ using **Equation 1**, with a K_{ZnEGTA} value of 3.78 × 10^8 M.[3,4]

$$K_{ZnEGTA}[Zn^{2+}]^2 + \left(1 + [EGTA]K_{ZnEGTA} - [Zn^{2+}]_t K_{ZnEGTA}\right)[Zn^{2+}] - [Zn^{2+}]_t = 0 \qquad \textbf{Equation 1}$$

The corresponding binding isotherm was then fit to **Equation 2** using a non-linear least squares iterative analysis in Python software. Titrations were repeated three times: the mean and standard error are reported in each case.

$$A = \frac{A_{min} + A_{max}K[M^{2+}]}{1 + K[M^{2+}]} \qquad \textbf{Equation 2}$$

^{1}H NMR Titration

S-APTRA was dissolved in D_2O (5 mL) to give a 1.14 mM solution according to an internal MeOH standard (0.5 μL). The ^{1}H NMR spectrum was recorded for S-APTRA with aliquots of the Zn^{2+} solution (which also contained S−APTRA to prevent ligand dilution over the course of the titration) gradually added until the concentration of Zn^{2+} surpassed the concentration of S-APTRA. In each case the solution was left to equilibrate for 5 min after the addition of the Zn^{2+} aliquots before acquisition of the spectrum.

Synthetic Intermediates

2-Nitrothiophenol

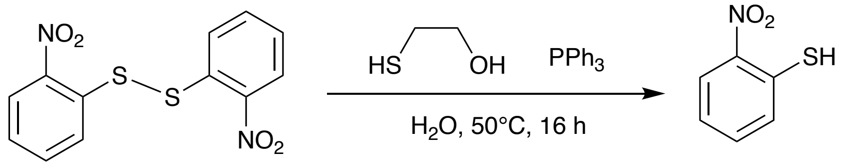

To a solution of 2-nitrophenoldisulfide (500 mg, 1.62 mmol) in THF (12.5 mL) was added PPh_3 (640 mg, 2.43 mmol, 1.5 equiv.), 2-mercaptoethanol (115 µL, 1.62 mmol, 1 equiv.) and water (0.3 mL). The resulting yellow suspension was heated at 50 ºC for 16 h. Upon cooling, the solvent was removed under reduced pressure. The residue was taken up into CH_2Cl_2, washed with brine, and dried over $MgSO_4$. Purification by column chromatography (gradient elution from hexane to 10% ethyl acetate in hexane) gave the title compound as a yellow solid (182 mg, 37%). R_f: 0.25 (silica, 10% ethyl acetate 90% hexane). ^{1}H NMR: (400 MHz, $CDCl_3$): δ_H 8.32 – 8.23 (1H, m, Ar H), 7.51 – 7.41 (2H, m, Ar H), 7.36 – 7.25 (1H, m, Ar H), 4.04 (1H, s, SH). Spectroscopic data are in agreement with previously reported data.[5]

Methyl(2-nitrophenyl)sulfane

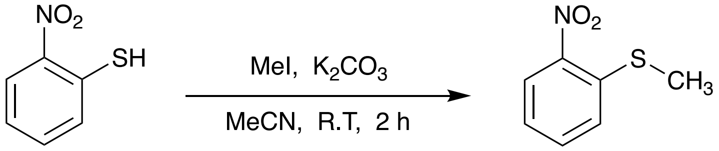

Potassium carbonate (484 mg, 3.51 mmol, 3 equiv.) was added to a solution of 2-nitrothiophenol (182 mg, 1.17 mmol) in anhydrous MeCN (3 mL) under N_2, giving a deep-red solution. Methyl iodide (80 µL, 1.29 mmol, 1.1 equiv.) was added, resulting in an almost instant colour change from red to yellow. The resulting suspension was stirred at room temperature for 2 h. The solvent was removed under reduced pressure, taken up into CH_2Cl_2, and filtered. The crude mixture was purified by column chromatography (hexane to 30% ethyl acetate in hexane) to give the title compound as a yellow solid (190 mg, 97%). ^{1}H NMR (400 MHz, $CDCl_3$): δ_H 8.28 (1H, dd, *J* 8.0, 1.5, Ar H), 7.61 (1H, ddd, *J* 8.0, 7.0, 1.5, Ar H), 7.44 – 7.37 (1H, m, Ar H), 7.32 – 7.23 (1H, m, Ar H), 2.53 (3H, s, C<u>H</u>$_3$). R_f: 0.2 (silica, 10% ethyl acetate 90% hexane). Spectroscopic data are in agreement with previously reported data.[6]

Methyl(2-aminophenyl)sulfane

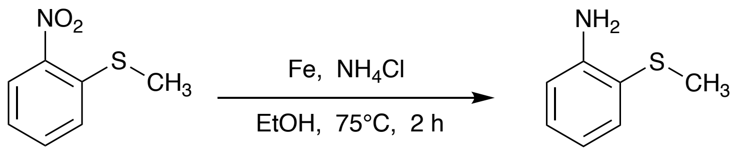

NH_4Cl (617 mg, 11.5 mmol, 2.5 equiv.) and iron powder (1.29 g, 23.1 mmol, 5 equiv.) were added to a solution of methyl(2-nitrophenyl)sulfane (780 mg, 4.62 mmol, 1 equiv.) in ethanol (5 mL) and water (5 mL). The solution was heated at 75 °C until the starting material had been fully consumed (monitored by TLC, typically around 2 h). The hot reaction mixture was filtered through celite, and the celite was flushed with ethanol (200 mL) to ensure that the product had fully passed through. The resulting filtrate was concentrated under reduced pressure to give a yellow residue, which was taken up into CH_2Cl_2 (30 mL) and poured into a separating funnel containing deionised water (30 mL). The organic layer was separated and the aqueous layer was extracted a further three times with CH_2Cl_2 (30 mL). The organic extracts were combined, dried over $MgSO_4$, and filtered. The solvent was removed under reduced pressure to give a dark green oil (490 mg, 76%), that was used in subsequent steps without further purification. ^{1}H NMR (400 MHz, $CDCl_3$): δ_H 7.40 – 7.35 (1H, m, Ar H), 7.16 – 7.07 (1H, m, Ar H), 6.78 – 6.70 (2H, m, Ar H), 4.29 (2H, br s, H^1), 2.38 (3H, s, C<u>H</u>3). R_f: 0.8 (silica, 30% ethyl acetate 70% hexane). Spectroscopic data are in agreement with previously reported data.[7]

Additional Binding Experiments

S-APTRA with Mg^{2+}

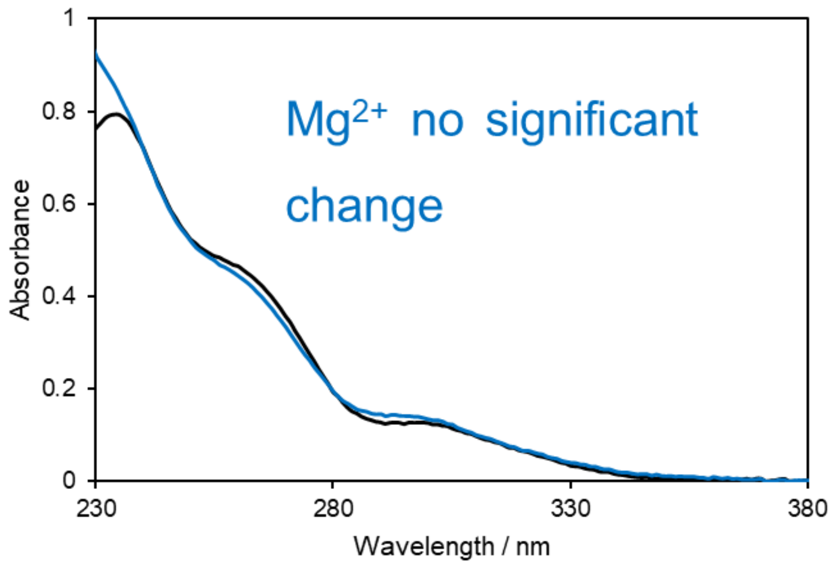

Fig. S1 *UV absorption spectrum of S-APTRA (50 μM) in the absence (black line) and in the presence of 1 M $MgCl_2$ (blue line) in buffered aqueous solution; [HEPES] = 50 mM, [KCl] = 100 mM, pH = 7.2, 295 K.*

S-APTRA with Ca^{2+}

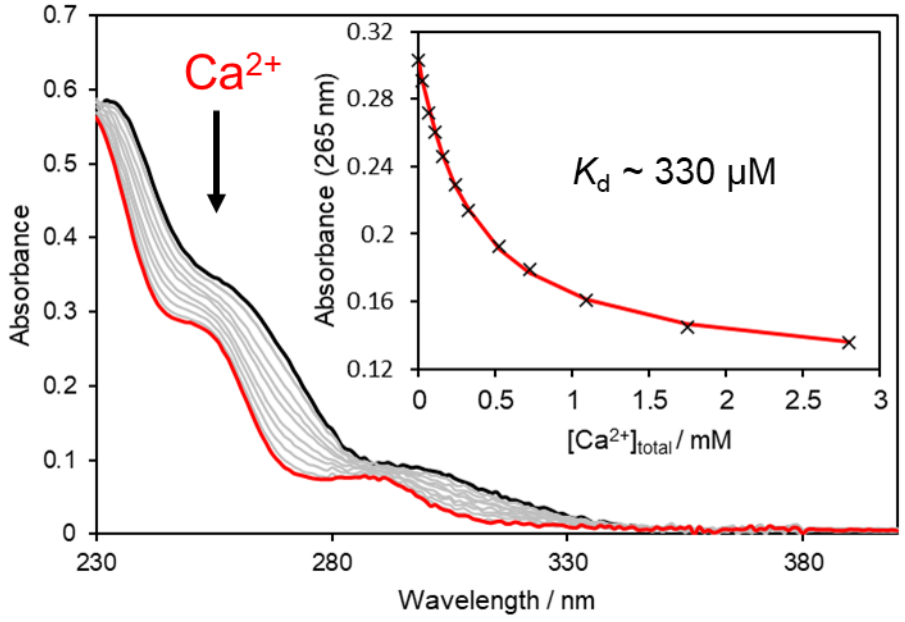

Fig. S2 *Representative UV absorption spectra of S-APTRA (50 μM) in the absence of Ca^{2+} (black line), increasing amounts of Ca^{2+} (grey lines) and near saturating amounts (red line) in buffered aqueous solution; [HEPES] = 50 mM, [KCl] = 100 mM, pH = 7.2, 295 K.*

Metal Competition Studies of S-APTRA

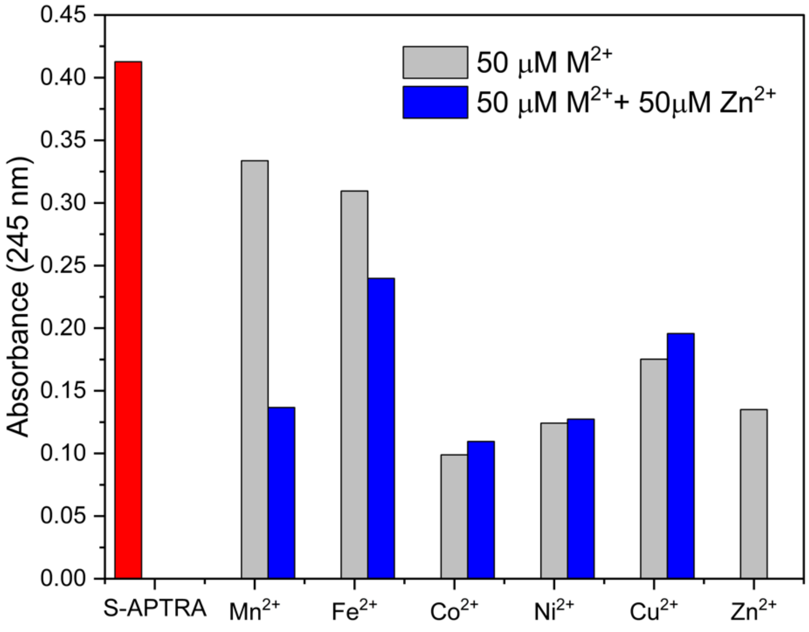

Fig. S3 *The absorbance of S-APTRA (concentration = 50 μM) at 245 nm in the presence of 50 μM of M^{2+} (grey bars) and 50 μM M^{2+} + 50 μM Zn^{2+} (blue bars), each as their chloride salts, in buffered aqueous solution; [HEPES] = 50 mM, [KCl] = 100 mM, pH = 7.2, 295 K.*

Job Plot of S-APTRA with Zn^{2+}

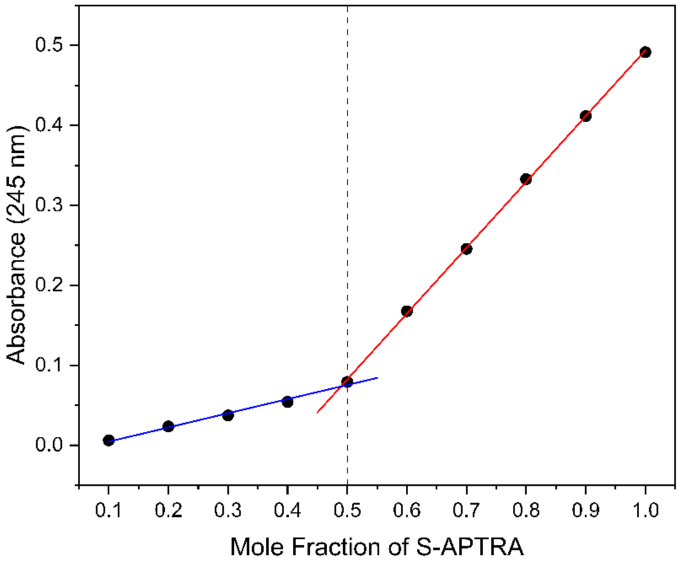

Fig. S4 *Job Plot of S-APTRA with Zn^{2+} showing 1:1 binding stoichiometry of the complex in buffered aqueous solution; [HEPES] = 50 mM, [KCl] = 100 mM, pH = 7.2, 295 K.*

^{1}H NMR Titration of S-APTRA with Zn^{2+}

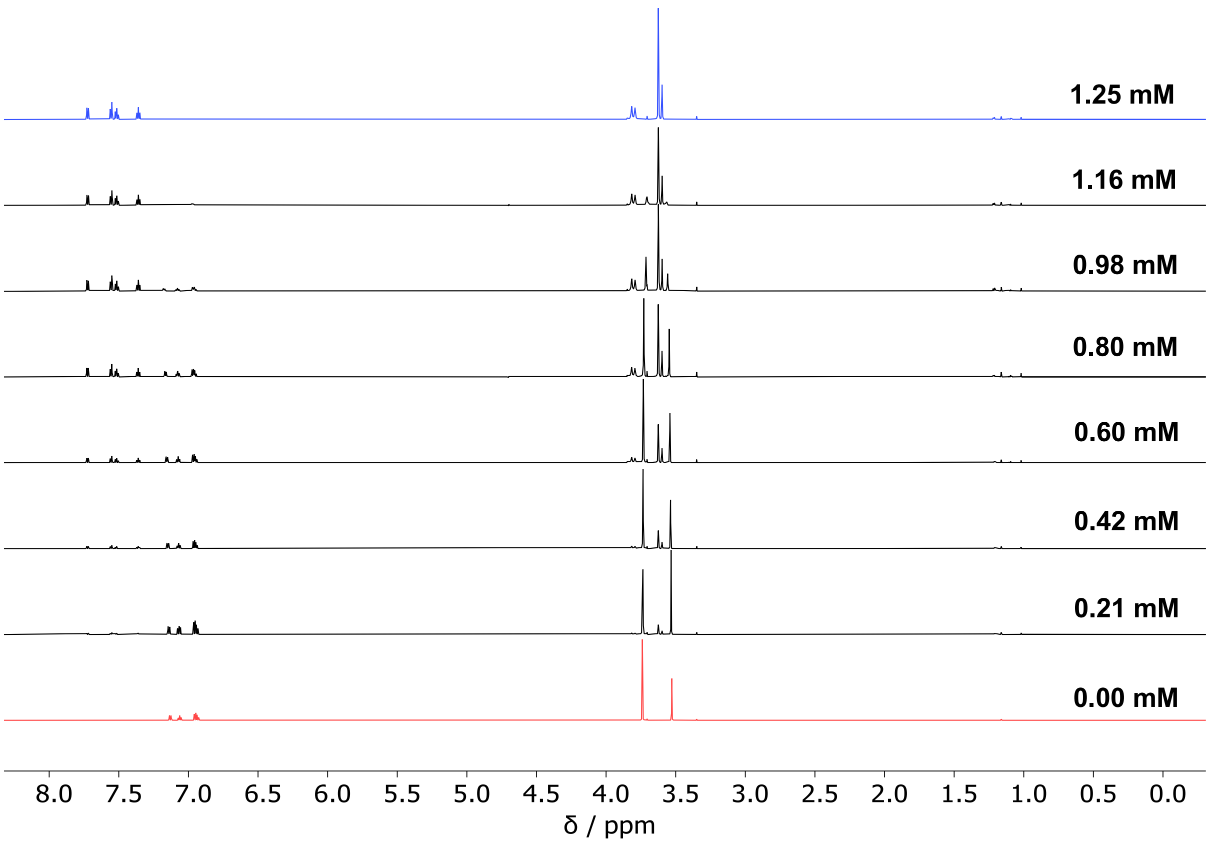

Fig. S5 *Full-range ^{1}H NMR spectra (600 MHz, 298 K) of S-APTRA (concentration of 1.14 mM in D_2O, with suppression of the H_2O signal), showing the change in the spectrum in the presence of the concentrations of Zn^{2+} indicated. See also Fig. 6 in the main text for expanded regions.*

S-APDIA with Mg^{2+}

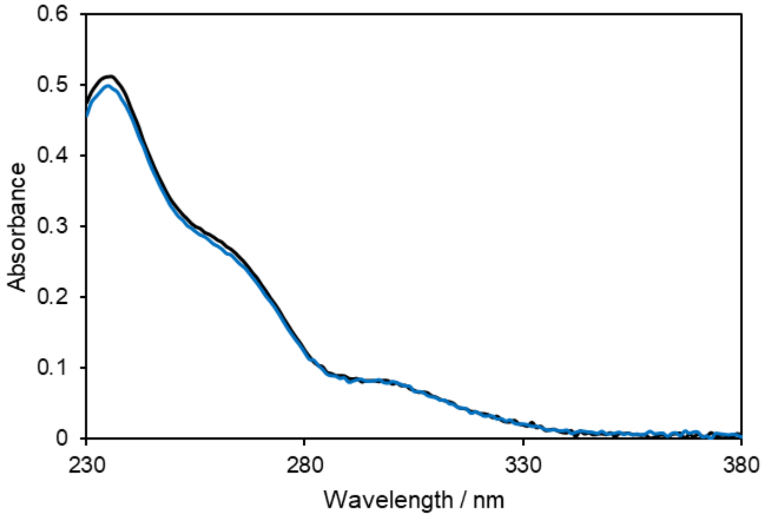

Fig. S6 *UV absorption spectra of S-APDIA (50 µM) in the absence of Mg^{2+} (black line) and in the presence of 25 mM $MgCl_2$ (blue line) in buffered aqueous solution; [HEPES] = 50 mM, [KCl] = 100 mM, pH = 7.2, 295 K.*

S-APDIA with Ca^{2+}

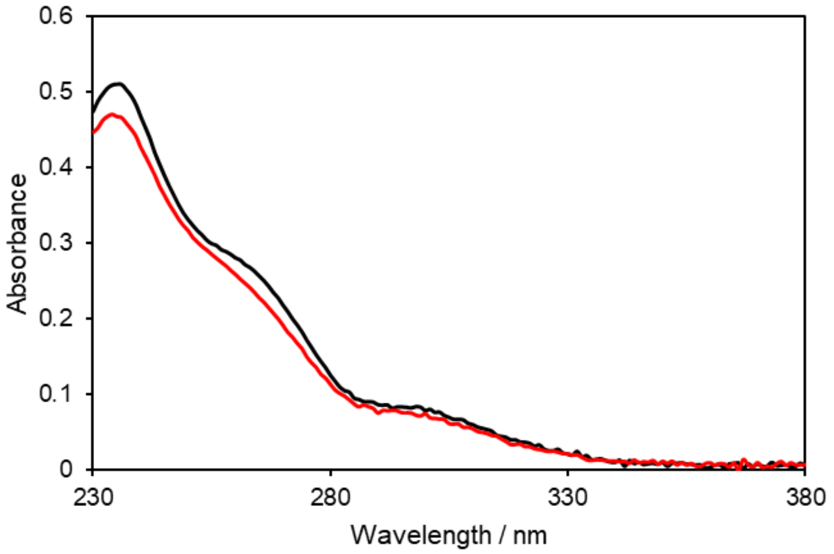

Fig. S7 *UV-VIS absorption spectrum of S-APDIA (50 µM) in the absence of* Ca^{2+} *(black line) and in the presence of 10 mM* $CaCl_2$ *(red line) in buffered aqueous solution; [HEPES] = 50 mM, [KCl] = 100 mM, pH = 7.2, 295 K.*

Job Plot of S-APDIA with Zn^{2+}

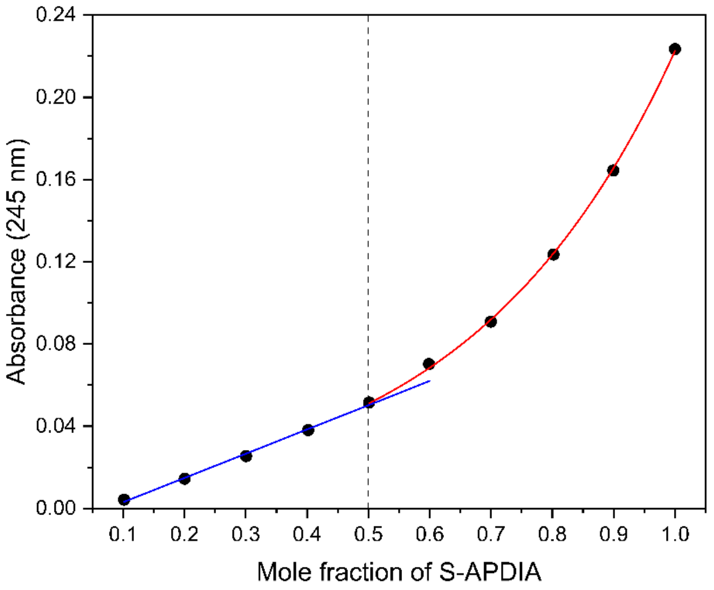

Fig. S8 *Job Plot of S-APDIA with* Zn^{2+}*, suggesting predominant 1:1 binding stoichiometry, in buffered aqueous solution; [HEPES] = 50 mM, [KCl] = 100 mM, pH = 7.2, 295K.*

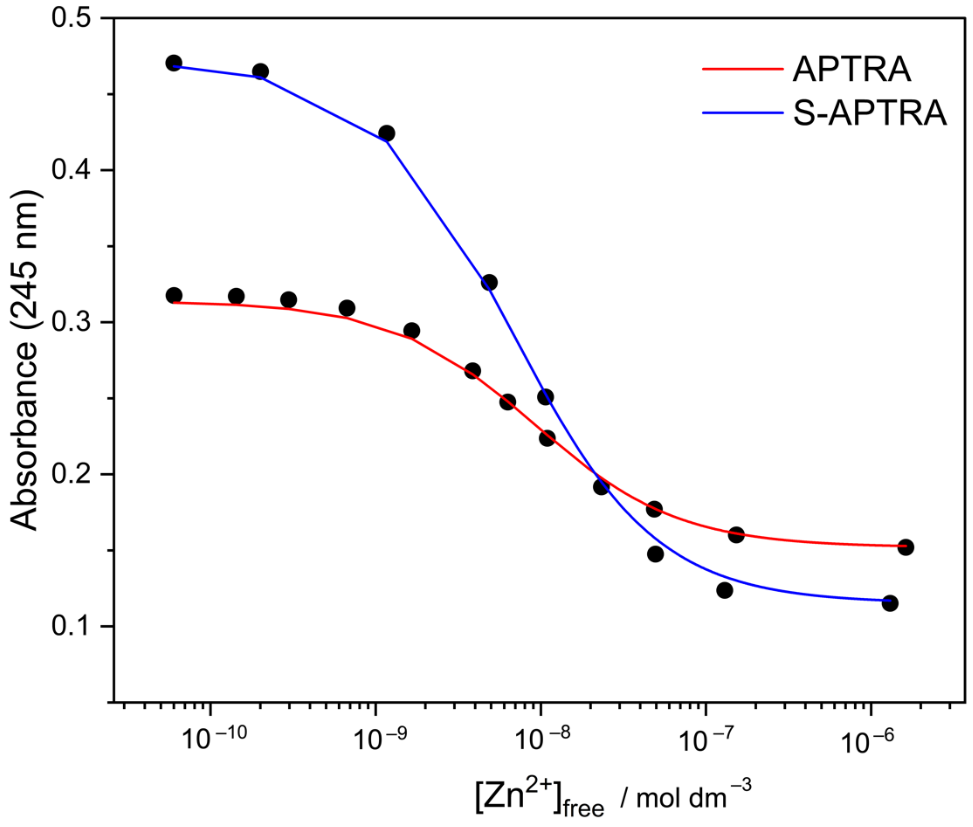

Fig. S9 *Overlaid zinc-binding plots for S-APTRA and APTRA in buffered aqueous solution (conditions as in Fig. 5 of the main text).*

^{1}H and ^{13}C NMR spectra of all new compounds reported

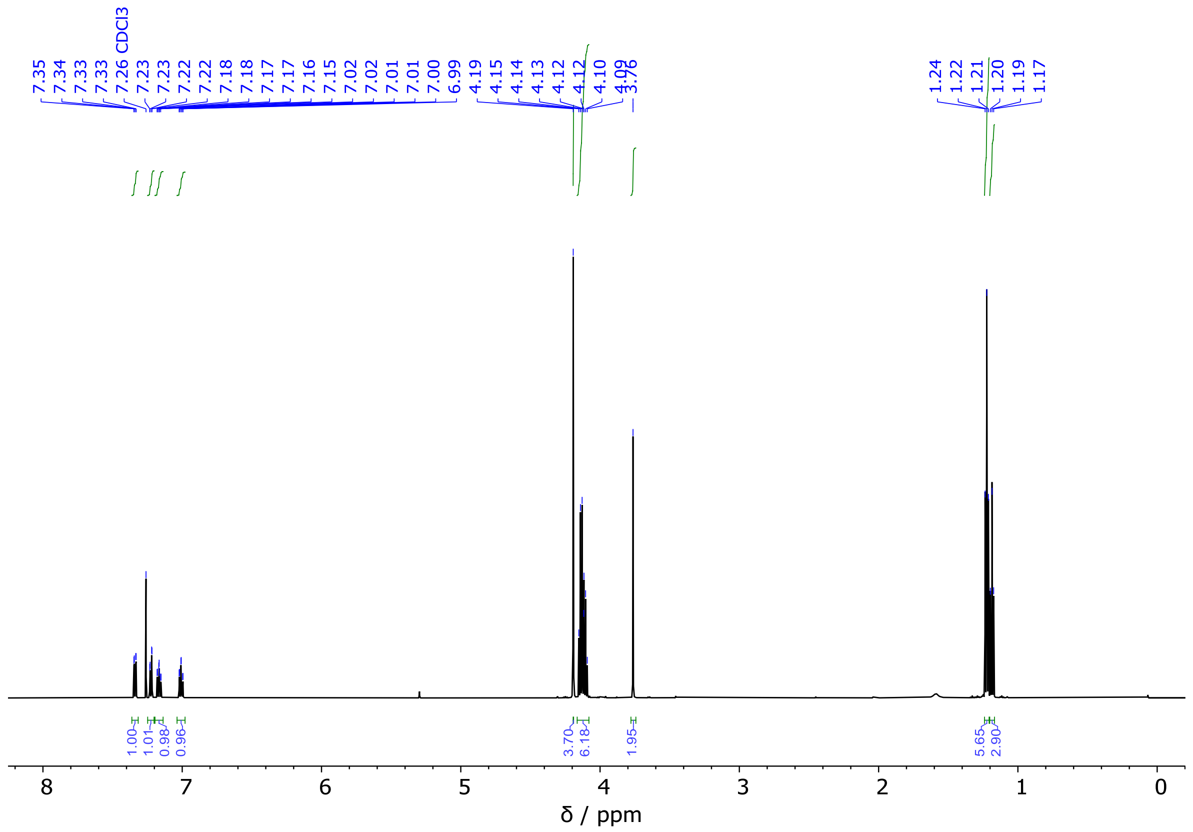

Fig. S10 *^{1}H NMR spectrum of S-APTRA-Et$_3$ in CDCl$_3$*

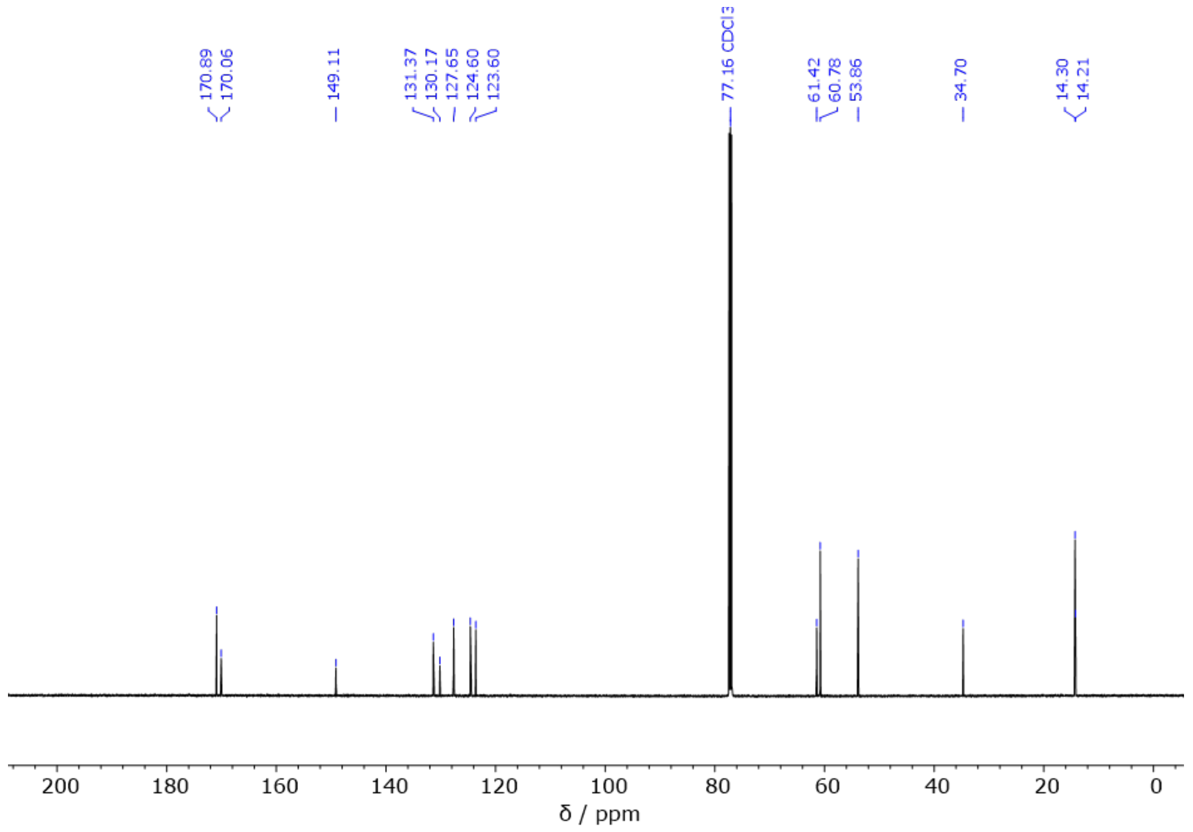

Fig. S11 *^{13}C NMR spectrum of S-APTRA-Et$_3$ in CDCl$_3$*

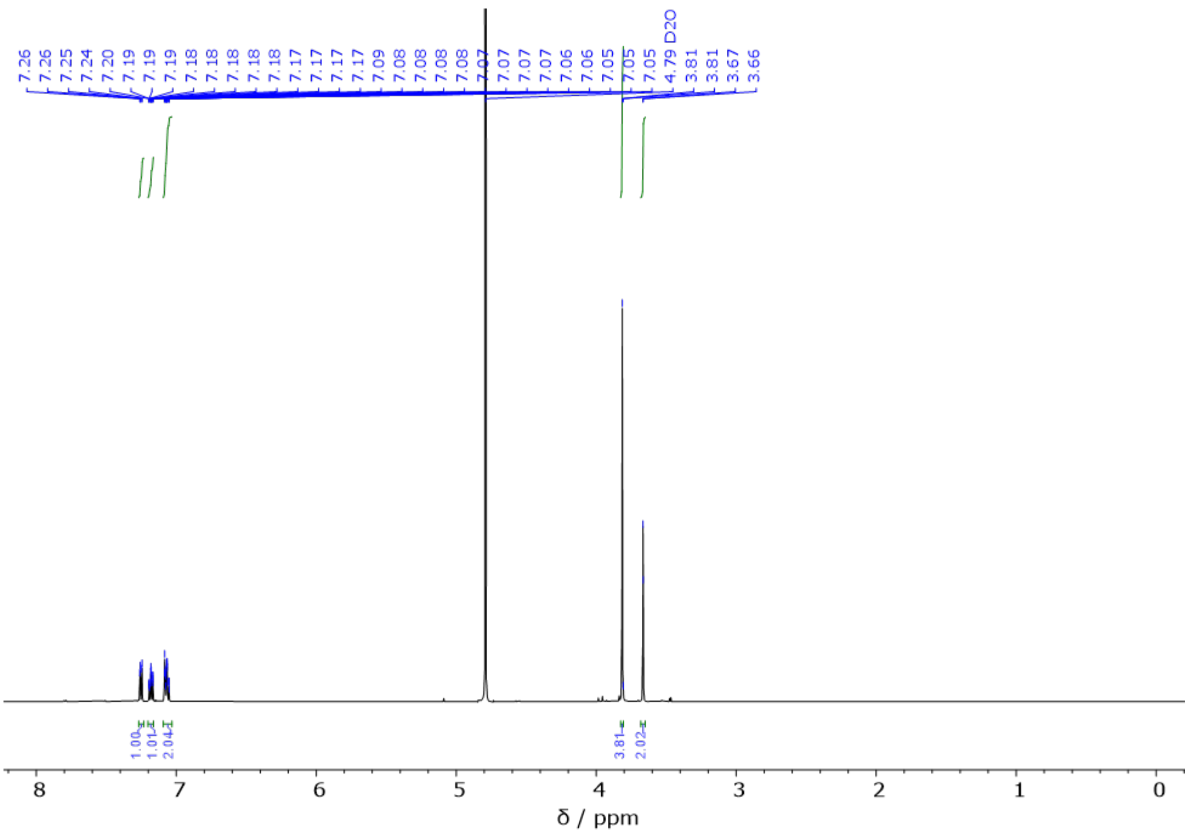

Fig. S12 *^{1}H NMR spectrum of S-APTRA in D_2O*

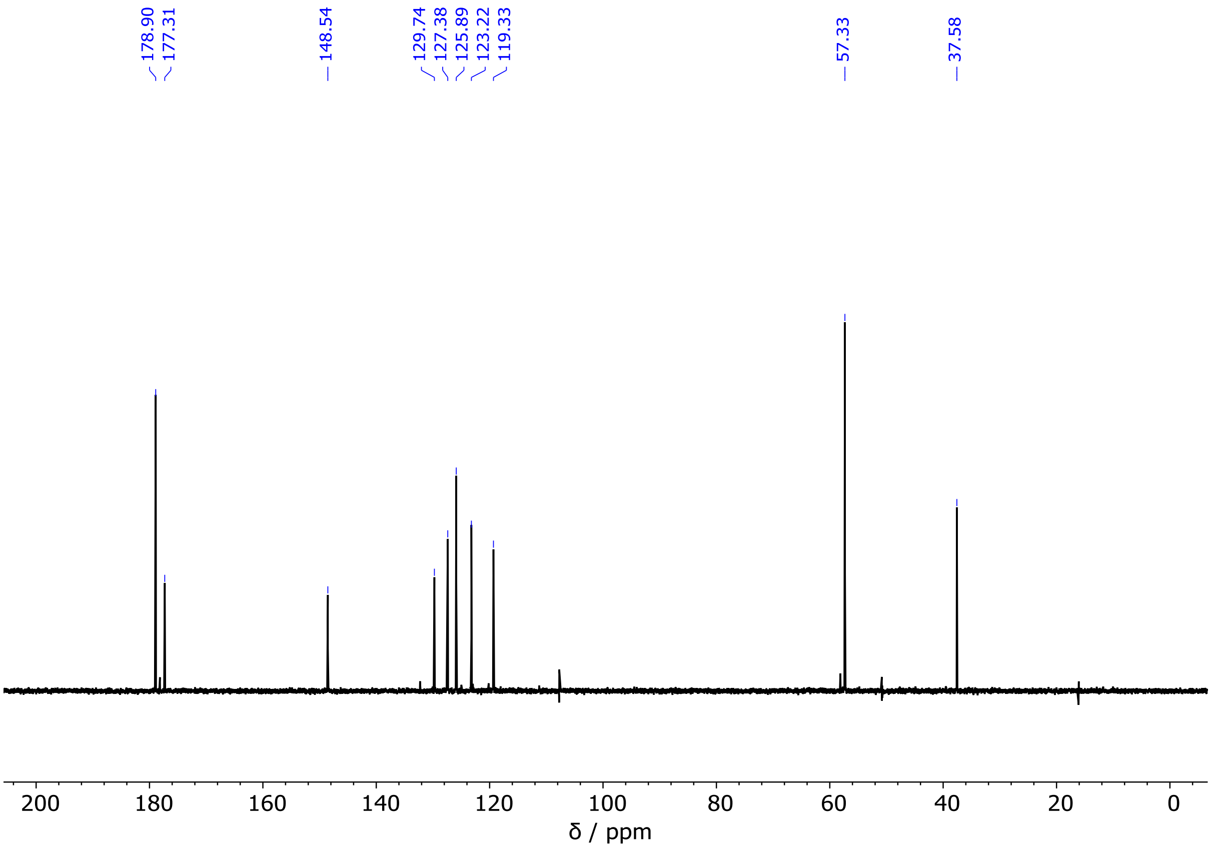

Fig. S13 *^{13}C NMR spectrum of S-APTRA in D_2O*

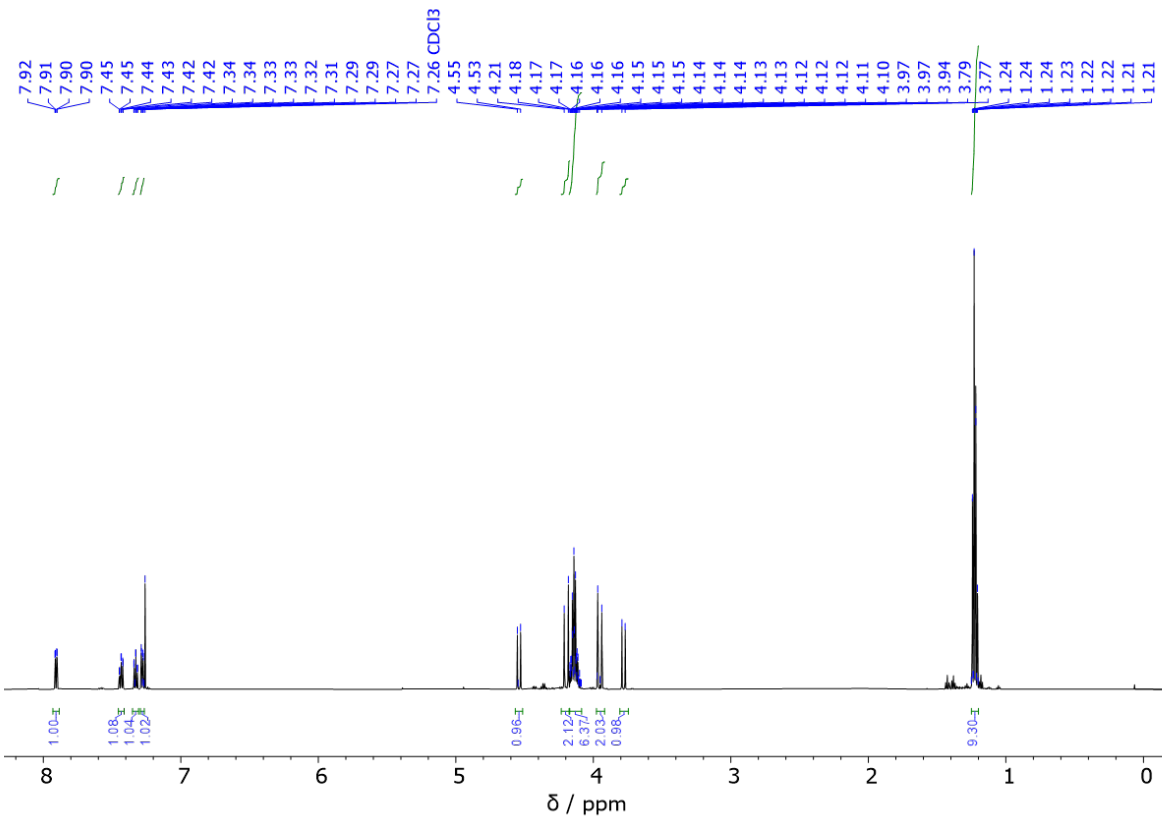

Fig. S14 *^{1}H NMR spectrum of SO-APTRA-Et$_3$ in CDCl$_3$*

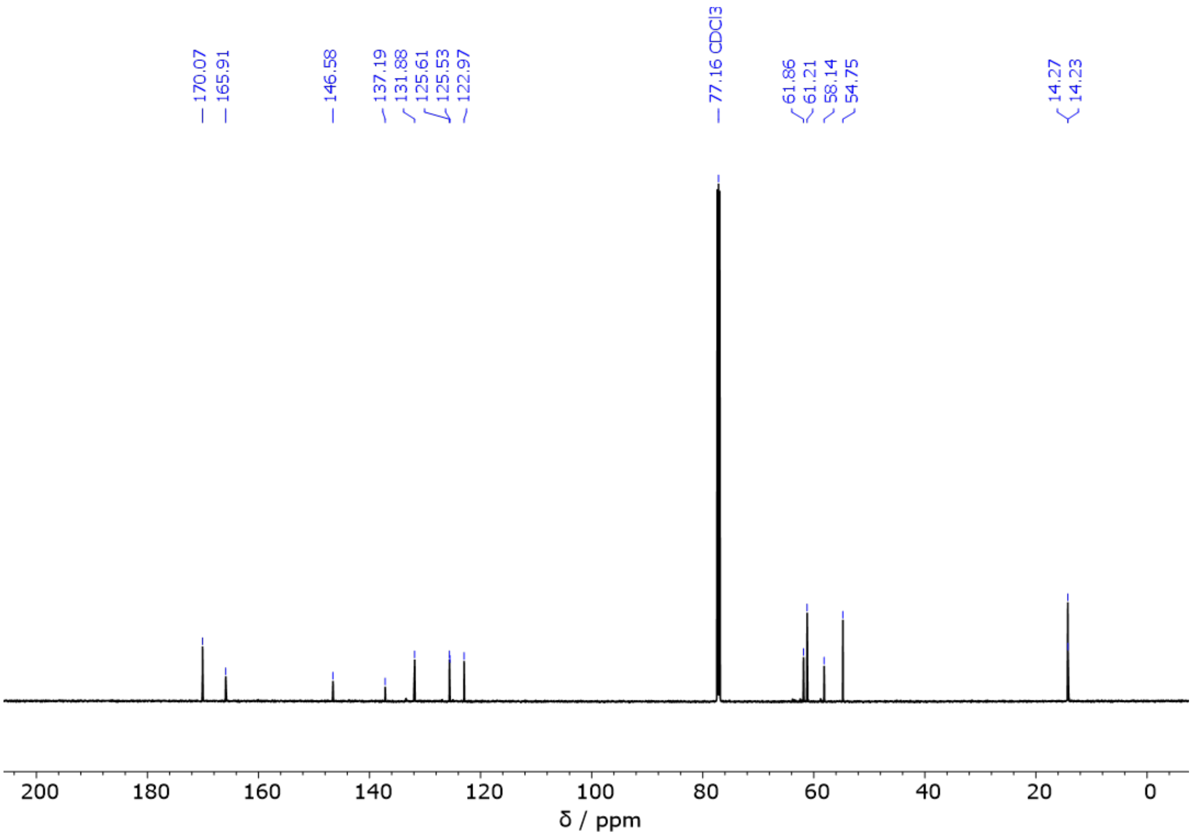

Fig. S15 *^{13}C NMR spectrum of SO-APTRA-Et$_3$ in CDCl$_3$*

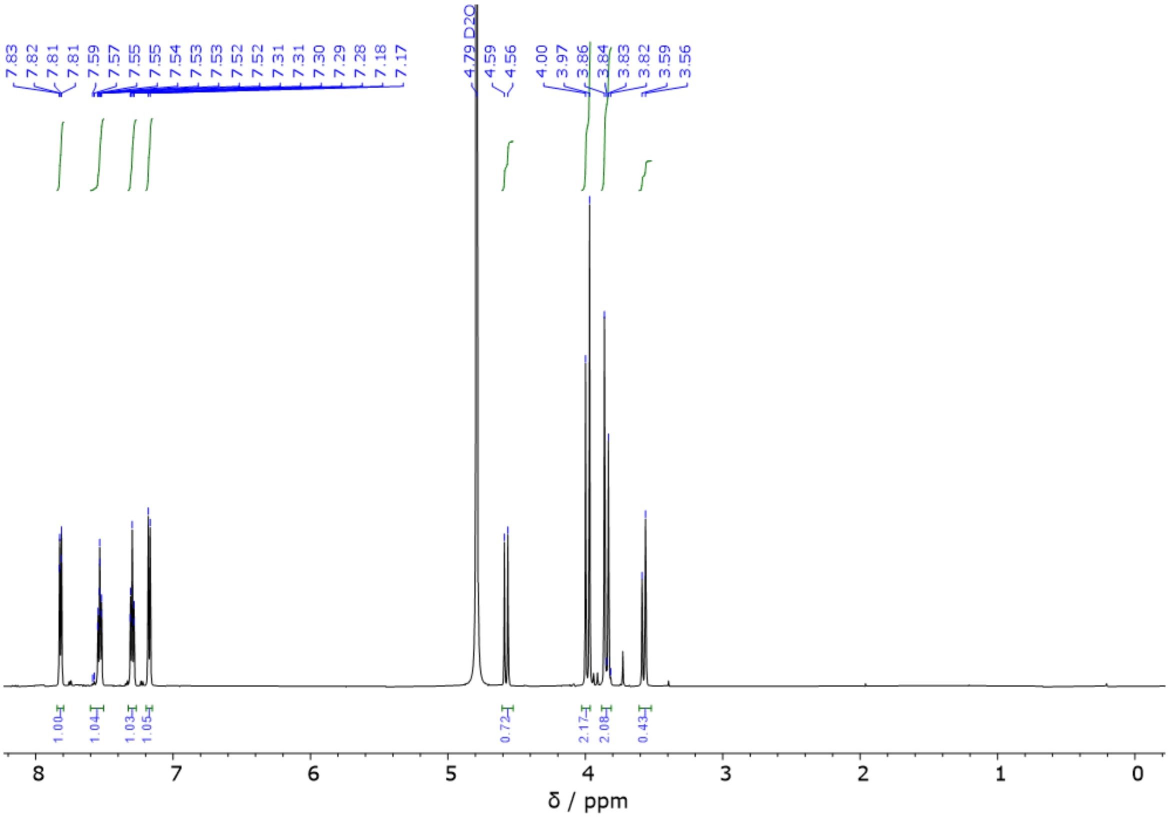

Fig. S16 *^{1}H NMR spectrum of SO-APTRA in D_2O*

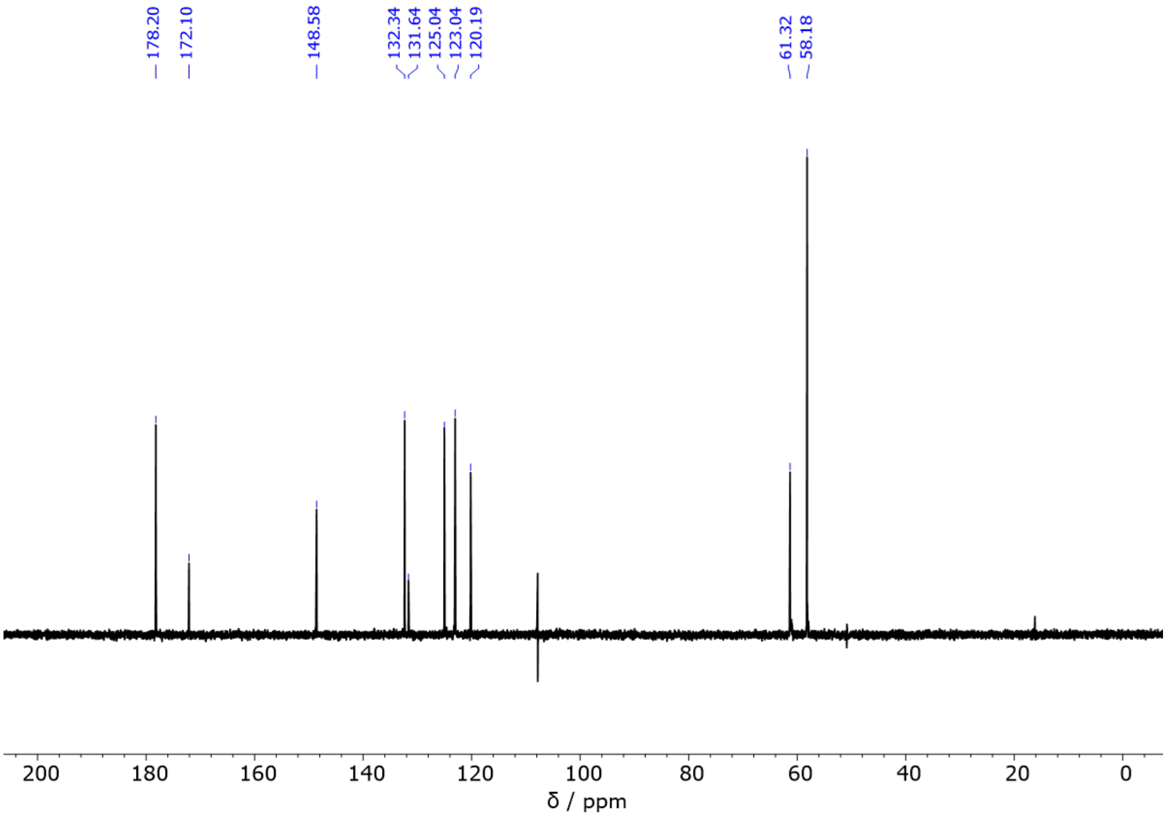

Fig. S17 *^{13}C NMR spectrum of SO-APTRA in D_2O*

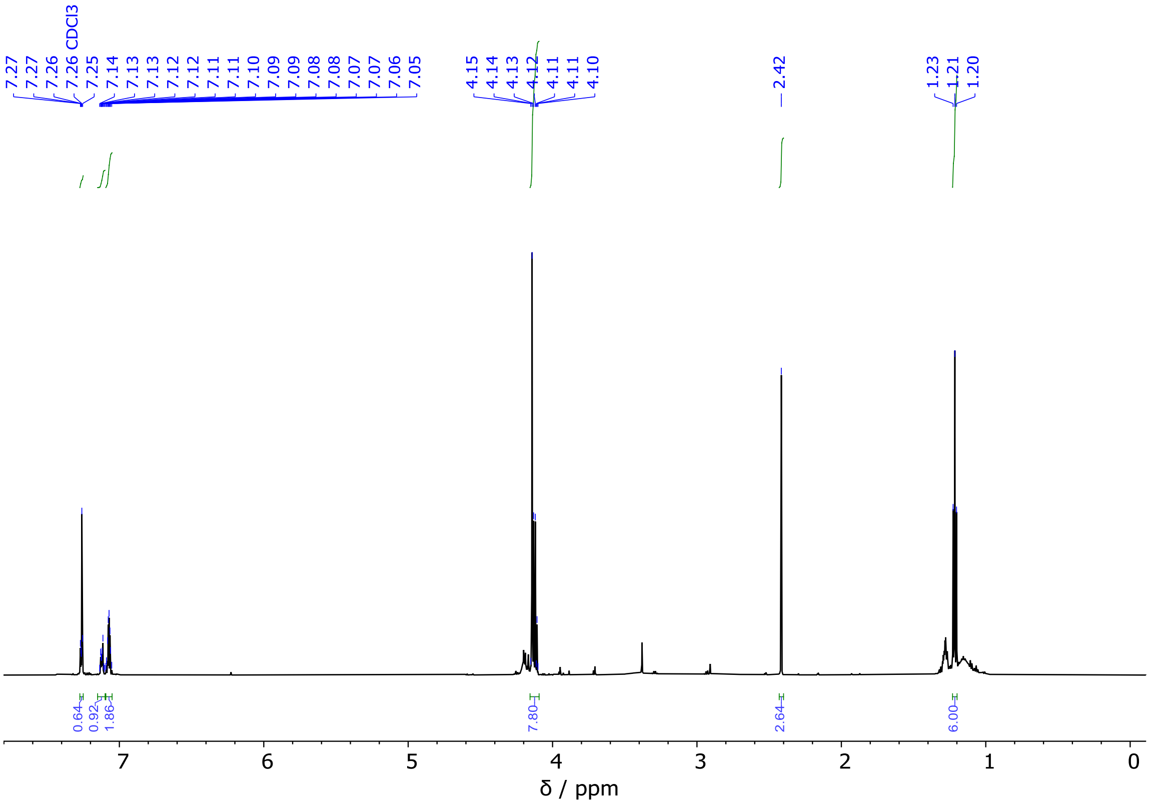

Fig. S18 *^{1}H NMR spectrum of S-APDIA-Et$_2$ in CDCl$_3$*

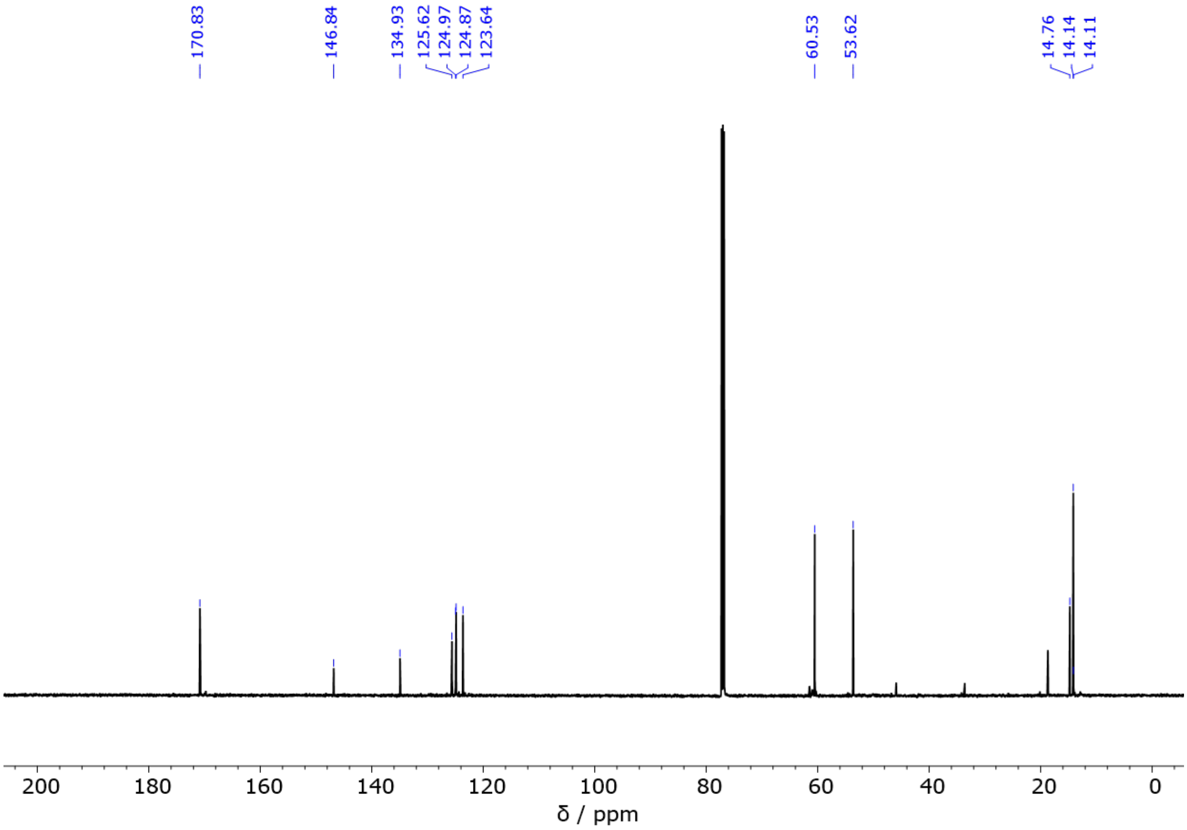

Fig. S19 *^{13}C NMR spectrum of S-APDIA-Et$_2$ in CDCl$_3$*

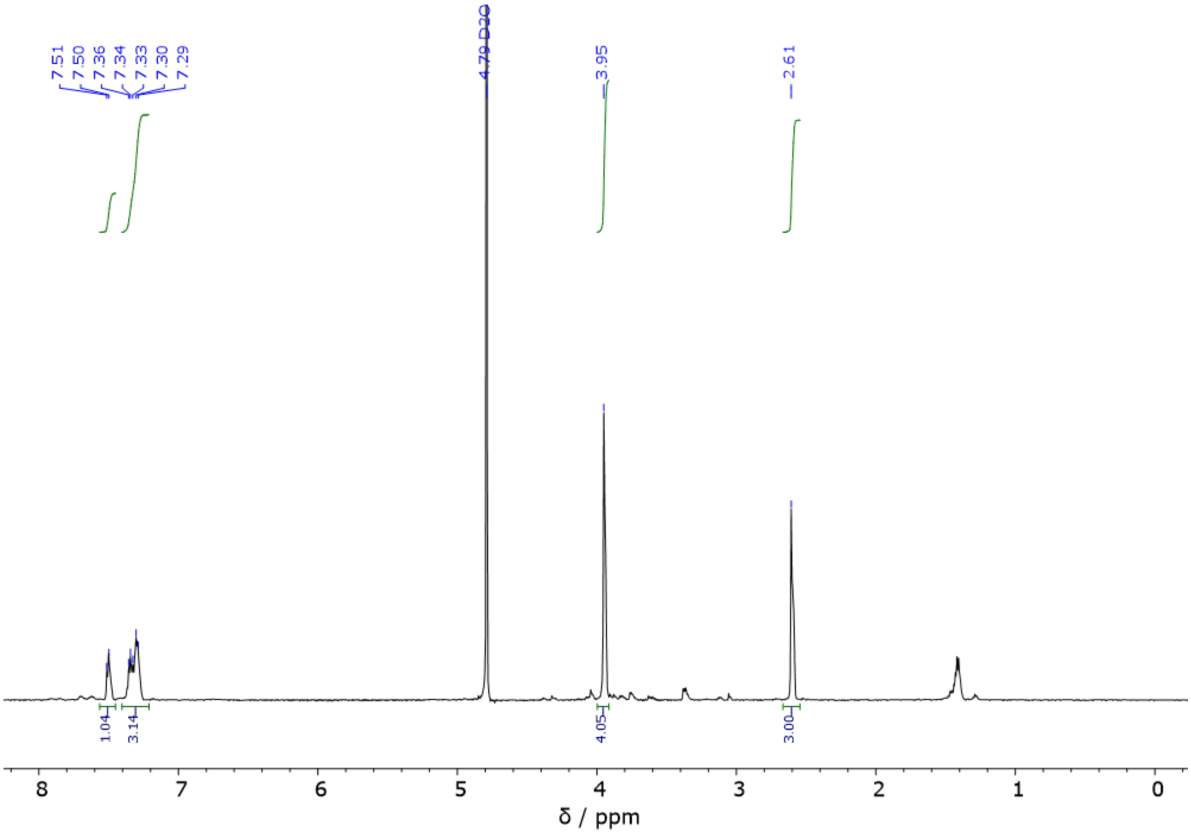

Fig. S20 *^{1}H NMR spectrum of S-APDIA in D_2O*

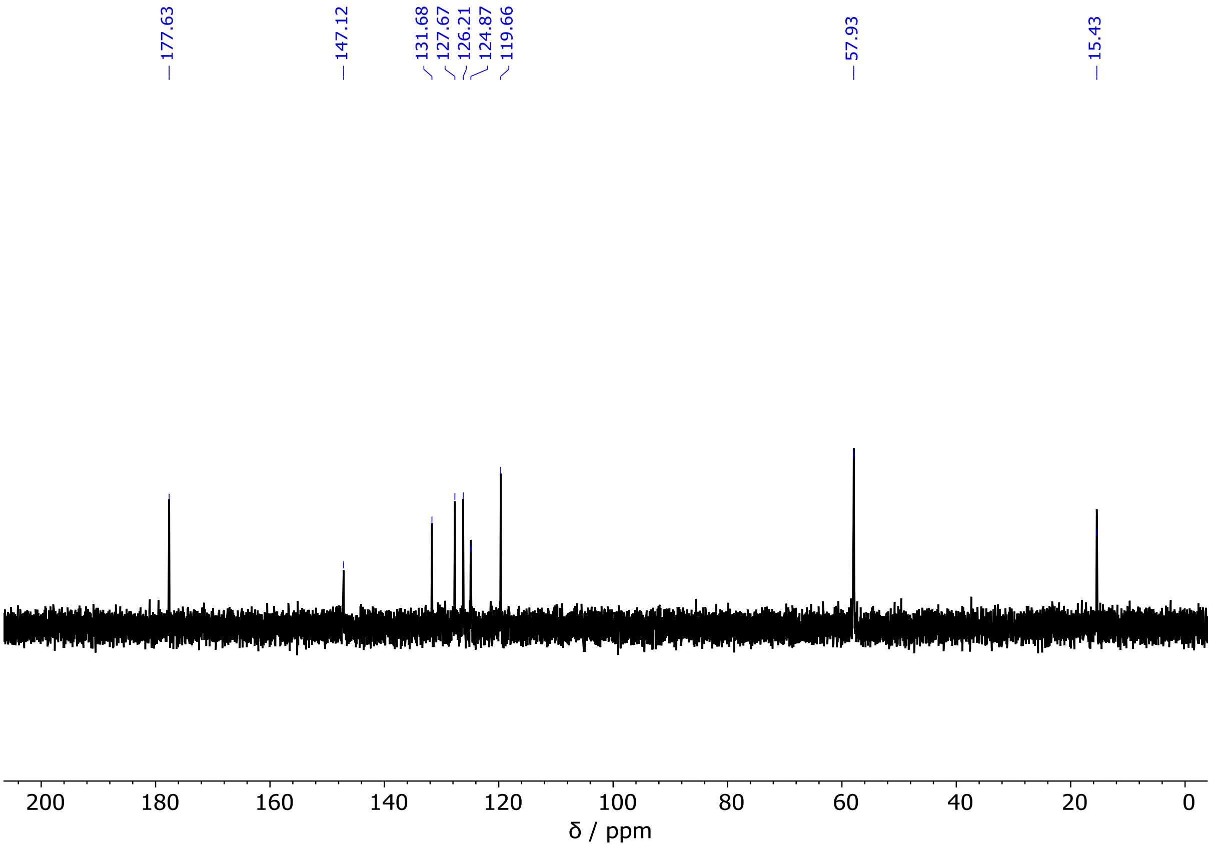

Fig. S21 *^{13}C NMR spectrum of S-APDIA in D_2O*

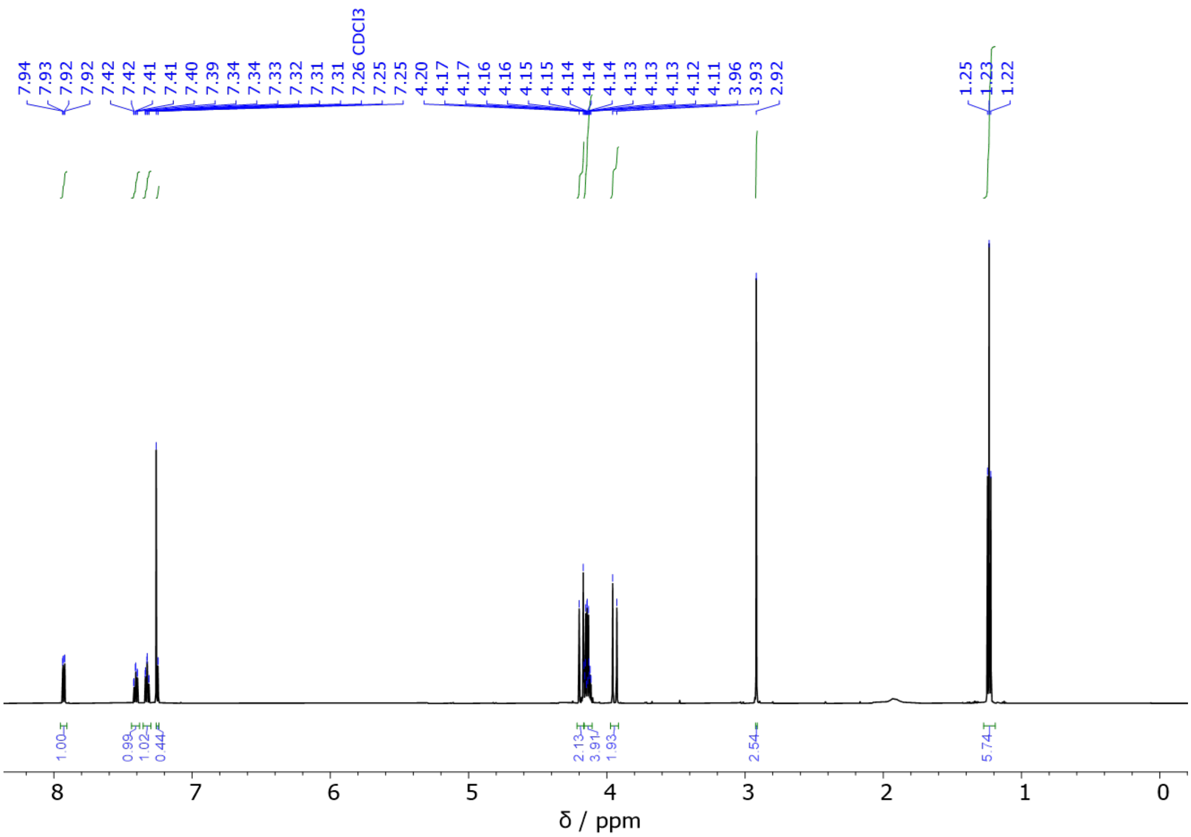

Fig. S22 *^{1}H NMR spectrum of SO-APDIA-Et$_2$ in CDCl$_3$. (One of the aromatic signals is partially obscured by residual CHCl$_3$).*

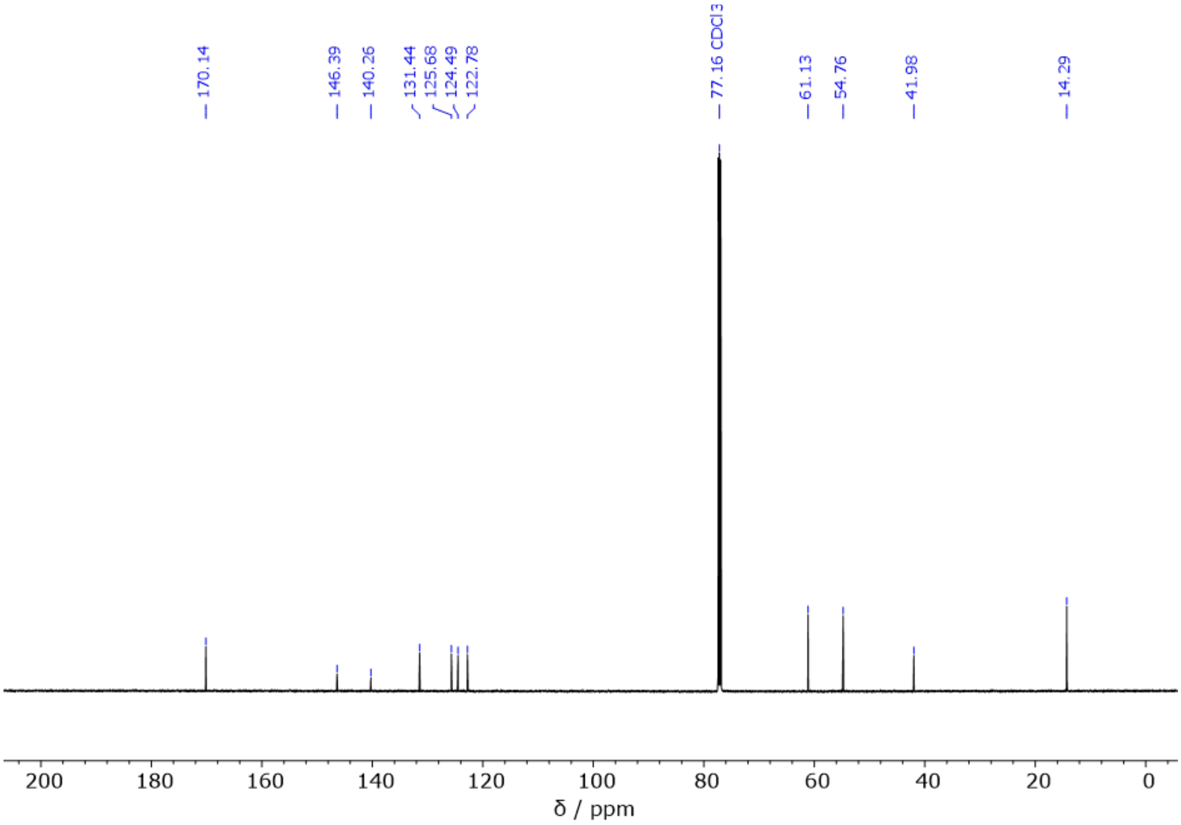

Fig. S23 *^{13}C NMR spectrum of SO-APDIA-Et$_2$ in CDCl$_3$.*

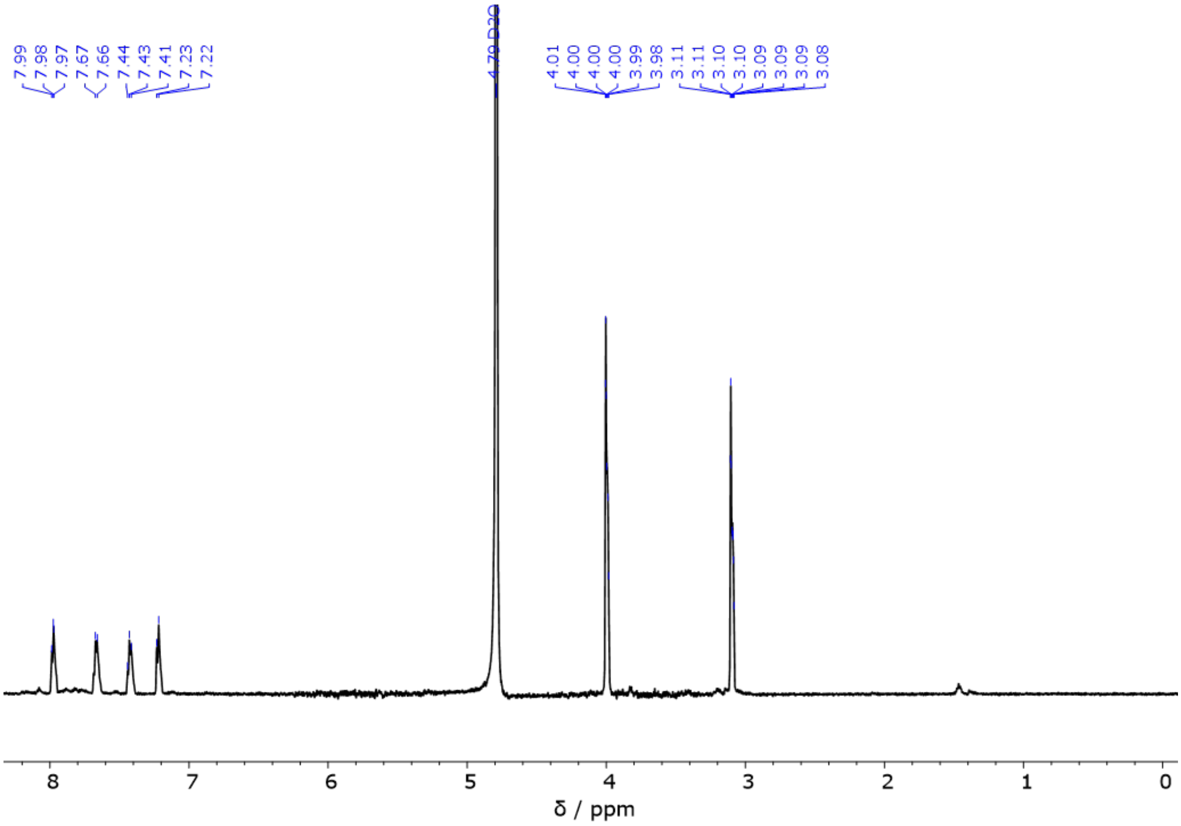

Fig. S24 *^{1}H NMR spectrum of SO-APDIA in D_2O*

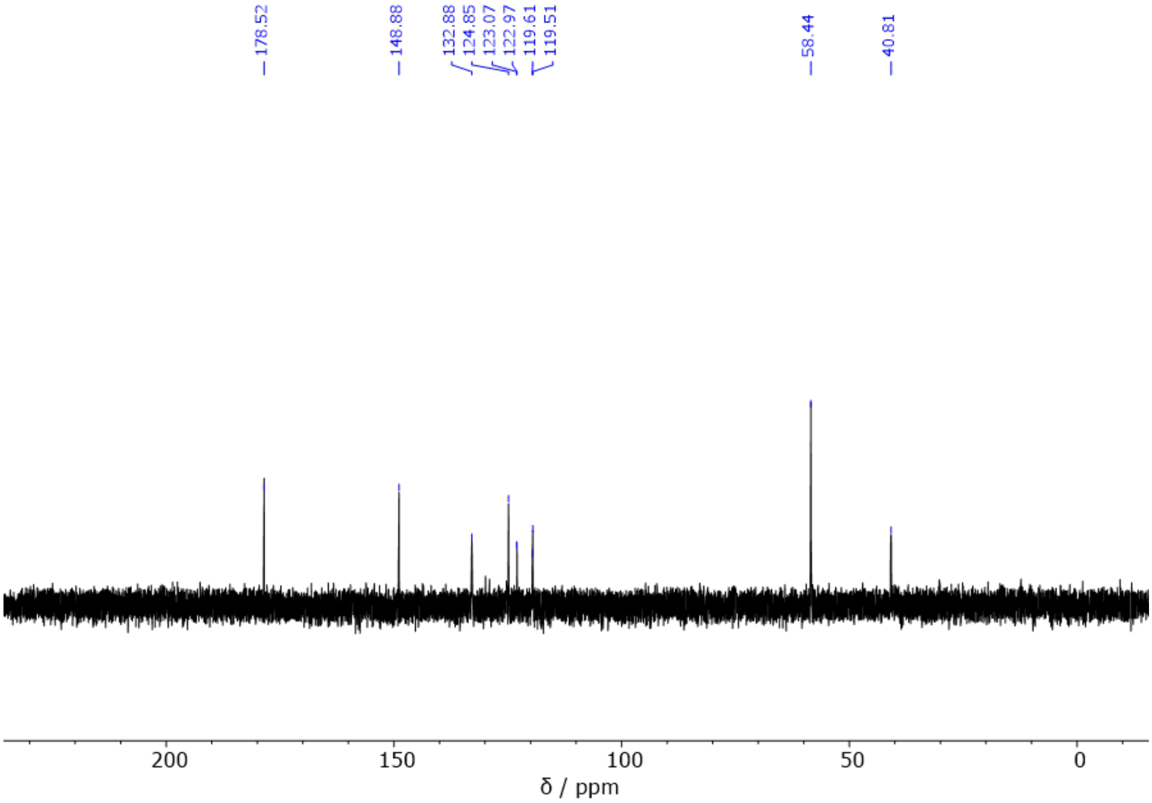

Fig. S25 *^{13}C NMR spectrum of SO-APDIA in D_2O*

References

1 P. Thordarson, Determining association constants from titration experiments in supramolecular chemistry. *Chem. Soc. Rev.*, 2011, **40**, 1305–1323.

2 P. Thordarson, Online tools for supramolecular chemistry research and analysis. www.supramolecular.org.

3 M. Brady, S. D. Piombo, C. Hu and D. Buccella, Structural and spectroscopic insight into the metal binding properties of the *o*-aminophenol-*N*,*N*,*O*-triacetic acid (APTRA) chelator: implications for design of metal indicator. *Dalton Trans.*, 2016, **45**, 12458–12464.

4 L. Fang, G. Trigiante, R. Crespo-Otero, C. S. Hawes, M. P. Philpott, C. R. Jones and M. Watkinson, Endoplasmic reticulum targeting fluorescent probes to image mobile Zn^{2+}. *Chem. Sci.*, 2019, **10**, 10881–10887.

5 R. R. Putta, S. Chun, S. H. Choi, S. B. Lee, D. C. Oh and S. Hong, Iron(0)-catalyzed transfer hydrogenative condensation of nitroarenes with alcohols: A straightforward approach to benzoxazoles, benzothiazoles, and benzimidazoles. *J. Org. Chem.*, 2020, **85**, 15396–15405.

6 J. Dong, J. Hu, X. Liu, S. Sun, L. Bao, M. Jia and X. Xu, Ionic reactivity of 2-isocyanoaryl thioethers: Access to 2-halo and 2-aminobenzthia/selenazoles. *J. Org. Chem.*, 2022, **87**, 2845–2852.

7 C. Gao and S. A. Blum, Silyl radical cascade cyclization of 2-isocyanothioanisole toward 2-silylbenzothiazoles through radical initiator-inhibitor symbiosis. *J. Org. Chem.*, 2022, **87**, 13124–13137.
